# Loop-extrusion and polymer phase-separation can co-exist at the single-molecule level to shape chromatin folding

**DOI:** 10.1038/s41467-022-31856-6

**Published:** 2022-07-13

**Authors:** Mattia Conte, Ehsan Irani, Andrea M. Chiariello, Alex Abraham, Simona Bianco, Andrea Esposito, Mario Nicodemi

**Affiliations:** 1 Dipartimento di Fisica, Università di Napoli Federico II, and INFN Napoli, Complesso Universitario di Monte Sant’Angelo, 80126 Naples, Italy; 2 Berlin Institute for Medical Systems Biology, Max-Delbrück Centre (MDC) for Molecular Medicine, Berlin, Germany; 3 Berlin Institute of Health (BIH), MDC-Berlin, Berlin, Germany

**Keywords:** Biological physics, Chromatin structure

## Abstract

Loop-extrusion and phase-separation have been proposed as mechanisms that shape chromosome spatial organization. It is unclear, however, how they perform relative to each other in explaining chromatin architecture data and whether they compete or co-exist at the single-molecule level. Here, we compare models of polymer physics based on loop-extrusion and phase-separation, as well as models where both mechanisms act simultaneously in a single molecule, against multiplexed FISH data available in human loci in IMR90 and HCT116 cells. We find that the different models recapitulate bulk Hi-C and average multiplexed microscopy data. Single-molecule chromatin conformations are also well captured, especially by phase-separation based models that better reflect the experimentally reported segregation in globules of the considered genomic loci and their cell-to-cell structural variability. Such a variability is consistent with two main concurrent causes: single-cell epigenetic heterogeneity and an intrinsic thermodynamic conformational degeneracy of folding. Overall, the model combining loop-extrusion and polymer phase-separation provides a very good description of the data, particularly higher-order contacts, showing that the two mechanisms can co-exist in shaping chromatin architecture in single cells.

## Abstract (web summary)

Two main mechanisms have been proposed to shape 3D genome architecture - loop extrusion and phase separation. Here the authors combine these mechanisms in polymer models in a manner that best fits 3D genome, based on both Hi-C and super-resolution locus imaging data, proposing that these two physical processes can indeed coexist simultaneously within cells to define loops and TADs.

## Introduction

To understand the molecular mechanisms that in the nucleus of cells establish the large scale 3-dimensional (3D) architecture of chromosomes^1–14^, encompassing DNA loops^15^, Topologically Associated Domains (TADs)^16,17^ and larger structures^13,18^, different physical processes have been proposed and investigated via chromatin models from physics^19–46^ and via computational approaches^47–61^. However, it remains unclear how well different mechanisms capture folding at the single molecule level, how they compare against each other in explaining experimental data and whether they compete or coexist. Here, we explore two recently discussed classes of models that focus on two distinct physical processes, respectively loop-extrusion and polymer phase-separation, that we compare against single-molecule super-resolution microscopy^6,62^ and bulk Hi-C data^15,63^ available in human loci in IMR90 and HCT116 cells.

Loop-extrusion and phase-separation based models reflect two classical, yet distinct scenarios of molecular biology to explain the formation of DNA contacts^64^. The first class considers the picture where physical proximity between distal sites is established by molecular motors that bind to DNA and extrude a loop^19,20,31,40,41^. This is an out-of-equilibrium, active physical process that involves energy, e.g., ATP, consumption. The model envisages that those loop-extruding complexes stochastically bind to a polymer chain and extrude loops until encountering another motor, an anchor site or unbinding from the chain. While the polymer becomes compacted in a linear array of loops, specific contacts are established between the motor anchor sites where extrusion halts, hence defining boundaries between subsequent chromatin regions. Experimental evidence indicates that Cohesin and Condensin can be components of the motor complex, while properly oriented CTCF sites can act as anchor points^41^. Computer simulations have shown that such a model can explain with good accuracy loops and TADs visible in bulk Hi-C contact maps^19,20,31,40,41^. Variants of such a model have been also developed where chromatin loops are formed by thermal random sliding of DNA into an extruding molecule^31^ or by, e.g., transcription-induced supercoiling^40^.

The second class of polymer models^21–30,32–39,42–46^ considers another classical scenario where physical proximity between distal DNA sites results from interactions mediated, for instance, by diffusing cognate bridging molecules, such as Transcription Factors, or from direct interactions produced, e.g., by DNA bound histone molecules. In the Strings and Binders (SBS) model^43,45^, for example, a chromatin filament is represented as a self-avoiding chain of beads, along which are located different types of binding sites for cognate diffusing binders that can bridge those sites. The binding sites have been correlated to different molecular and epigenetic factors, ranging from active and poised Pol-II to eu- and heterochromatin sites^21,27,28,46^. The steady-state 3D conformations of the system are determined by the laws of physics and fall in different structural classes corresponding to its thermodynamics phases. In the SBS model, for instance, upon increasing the concentration or affinity of binders, the system undergoes a polymer phase-separation transition from a coil, i.e., randomly folded, to a globular state, where distinct globules self-assemble along the chain by the interactions of cognate binding sites^24,27,35,45^. Polymer physics explains that the thermodynamic phases are independent of the specific origin of the interactions—e.g., direct or mediated by diffusing factors - so different models can belong to the same universality class^65^. For that reason, the thermodynamic phases of, say, the SBS model also occur in models with direct chromatin interactions. Those phase transitions result in structural changes of the chain that spontaneously establish contact or segregation of specific, distal sites, such as genes and their regulators. Such a class of models has been shown to explain Hi-C, SPRITE, GAM and microscopy contact data across the genome, from the sub-TAD to chromosomal scales^21–30,32–38,42–46^, also at the single molecule level^35,38^.

It is unclear, however, how loop-extrusion and polymer phase-separation perform relative to each other in capturing chromatin folding and whether they compete or coexist in establishing chromosome architecture in single cells. Here, we implemented different versions of those models to benchmark their structural predictions at the single-molecule level against independent multiplexed FISH data available in specific genomic loci^6^. We simulated first a simple loop-extrusion (LE) model^20^ of those loci. Next, we developed an extended LE (eLE) model where, to mimic epigenetic differences of single cells, the anchor sites can differ across single molecules^29^. Additionally, in the eLE model the genomic locations of the anchor sites, and their single-molecule presence probability, are optimized to best fit experimental contact data. We also considered the SBS model of the studied loci^35^ and, finally, we introduced a model combining eLE and SBS (the LE+SBS model), i.e., a model where in a single molecule both LE and SBS mechanisms act simultaneously. We find that both loop-extrusion and phase-separation based models can quantitatively explain ensemble-averaged microscopy and bulk Hi-C data, albeit the simple LE model is only partially effective. Our single-molecule analyses show that both types of models do capture the main features of single-cell chromatin conformations and higher-order contacts. Yet, phase-separation based models better reflect the experimentally reported segregation in globules of the considered genomic loci and their cell-to-cell structural variability. Such a variability results from two main concurrent sources: the intrinsic thermodynamic degeneracy of polymer folding and single-cell epigenetic heterogeneity. Consistent with such a picture, the LE+SBS model turns out to provide overall an excellent description of all the different datasets and to have the least discrepancy with microscopy triple contact data, supporting the view that loop-extrusion and phase-separation can coexist at the single-molecule level in determining chromatin architecture.

## Results

### Polymer models of the studied loci

**Fig. 1:**
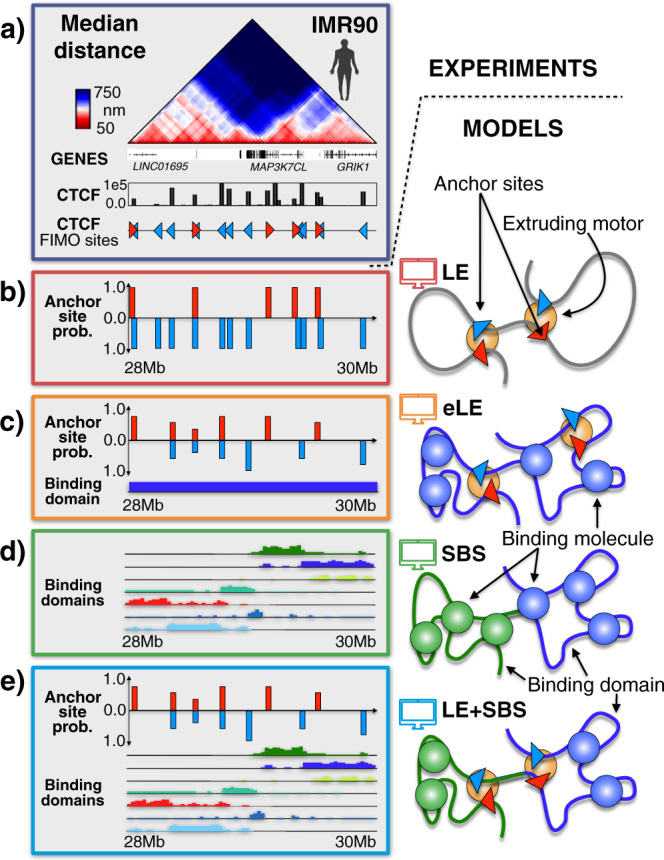
Scheme of the investigated polymer models. We used Molecular Dynamics simulations to investigate polymer models where folding is based on two different physical processes: (i) DNA loop-extrusion and (ii) polymer phase-separation, recapitulated respectively by the LE^19,20^ and by the SBS models^43,45^. **a** Microscopy median distance^6^ and ENCODE^67^ CTCF data are shown for the studied 2 Mb wide locus in human IMR90 cells. **b** We considered a simple Loop-Extrusion (LE) model^20^ where active motors extrude polymer loops until encountering another motor or CTCF anchor points with opposite orientation, which are fixed and equal in all single-molecule simulations (anchor probability = 1). **c** We also considered an extended version of the LE (eLE) whose anchor site locations are optimized, independently of CTCF, to best reproduce Hi-C and average microscopy data. To represent the epigenetic heterogeneity of single cells, those anchor sites have a finite probability to be present in a model single molecule^29^. **d** In the Strings and Binders (SBS) model^35^, a chromatin filament is represented as a self-avoiding chain of beads including different types of binding sites (colors) for diffusing cognate binders that can bridge those sites. The model undergoes a phase-separation of the chain in distinct globules^35^. The binding site locations are determined by the PRISMR method and correlate with different combinations of chromatin architecture factors including, but not limited to, CTCF and cohesin^28,35^. **e** We also considered a polymer model (LE + SBS) where in a single molecule both the eLE and SBS mechanisms act simultaneously.

We implemented the polymer models of two 2 Mb wide genomic loci in human IMR90 and HCT116 cells where, as stated, single-cell super-resolution microscopy data^6^ are available at 30 kb resolution (Fig. 1a, Supplementary Fig. 1a). To assess the role of the different ingredients of the models, we developed distinct versions that we compared against single-cell data.

First, we implemented a simple LE model^20^, where loop-extruding motors stochastically bind to a polymer bead chain and extrude loops until encountering anchor points with opposite orientation or another motor or unbinding from the chain (Fig. 1b and Methods). The position and orientation of the anchor points are identified by the FIMO standard motif finding analysis^66^ based on the peaks of CTCF ChIP-seq data from ENCODE^67^. While the motors can stochastically bind to and unbind from the chain, the anchor sites are fixed and equal in all single-molecule computer simulations. Their anchoring strength is set to 100%, i.e., when an extruder arrives at an anchor point it remains blocked at that position, yet we checked that the overall results do not change for strengths in the range down to 60% (Methods). This model is hereafter referred to as the LE model.

To explore the potential of the loop-extrusion mechanism and to dissect the roles of its ingredients beyond such a minimal implementation, we also considered a more refined version, hereafter named the extended LE (in short, eLE). In the eLE model, to mimic epigenetic differences across single cells, each anchor site is present, with a given probability, only in a subset of model single-molecules^29^ (Fig. 1c, Supplementary Fig. 1b). Additionally, to best fit average contact and microscopy distance data, we searched for the optimal genomic location and probability of the motor anchor sites, independently of CTCF tracks (Methods). Finally, to better form TADs and globules (see below), the beads of the polymer chain are subject to a self-interaction produced by unspecific bridging molecules. In our studied loci, we found that the probability to be present in a model single-molecule is different for different anchor sites, ranging from roughly 50–100% (Fig. 1c, Supplementary Fig. 1b), a range consistent with current estimates of cell epigenetic heterogeneity^68^. Also, interestingly, while the optimized eLE sites are all CTCF sites, not all LE CTCF sites are retained in the model after the optimization because a fraction of them (roughly 50%) is found to be redundant in the eLE (i.e., not required to better explain contact data, Supplementary Figs. 2a, b, 3a, b and Methods). Additionally, while the eLE anchor sites are all characterized by CTCF/cohesin signatures, they also have overlaps with different chromatin marks such as H3K27ac, H3K4me1, H3K27me3 and Pol-II (Supplementary Figs. 2c, 3c), supporting the view that in the genome not all CTCF sites are equivalent, as reported in recent experiments^69,70^, and that they could be combined with other signals in determining loop sites.

Next, in the considered loci we implemented the SBS model^35^ whereby chromatin is represented as a self-avoiding chain of beads, in a thermal bath, with specific binding sites for cognate diffusing molecular binders (Fig. 1d, Supplementary Fig. 1c and Methods). The location and types of binding sites are different for the different loci and are inferred via a machine learning procedure based on the PRISMR method, which takes as input only Hi-C data^28,35^. The model of the HCT116 locus has four binding site types and the model of the IMR90 locus has seven types, each visually represented by a different color along the chain (Fig. 1d, Supplementary Fig. 1c). The binding site types have been shown to correlate with different combinations of chromatin architectural factors, such as CTCF/cohesin, H3K4me3 or H3K4me1^35^. As mentioned above, the equilibrium 3D conformations of the SBS model fall in structural classes corresponding to its thermodynamics phases^65^: upon increasing binder concentration or affinity above a threshold value, the system undergoes a phase transition from a coil (i.e., randomly folded) to a polymer phase-separated state where distinct, compact globules self-assemble along the chain in correspondence of its different, prevailing binding domains (i.e., locally enriched colors)^35^. The intrinsic thermodynamic degeneracy of the states of the model results in a broad variety of 3D single-molecule conformations^35^ (Methods). We also developed a variant of the SBS model where cognate DNA sites have direct physical interactions, rather than mediated by binders, and our overall findings remain unchanged (Supplementary Fig. 4, Methods) as expected from Statistical Mechanics^65^.

Finally, to check whether active mechanisms, such as loop-extrusion, and passive mechanisms, such as thermodynamic polymer phase-separation, could coexist to shape chromatin architecture in the studied loci, we implemented a polymer model combining the above described eLE and SBS models, i.e., a model where both mechanisms act simultaneously in each single molecule (named the LE+SBS model, Fig. 1e, Supplementary Fig. 1d and Methods). For each of the considered models, an ensemble of 3D conformations was obtained via Molecular Dynamics simulations in the steady state^29,35^ (Methods). In all the considered cases, the model unit length scale was mapped into physical units by equating the median gyration radius to its corresponding experimental counterpart^6,35^ (Supplementary Fig. 5, Methods).

### Both loop-extrusion and phase-separation based models recapitulate average microscopy and Hi-C data

To benchmark the different models, we focused first on how they recapitulate population-averaged experimental data by comparing their median distance and contact maps against, respectively, multiplexed FISH^6^ and bulk Hi-C data^15,63^.

**Fig. 2:**
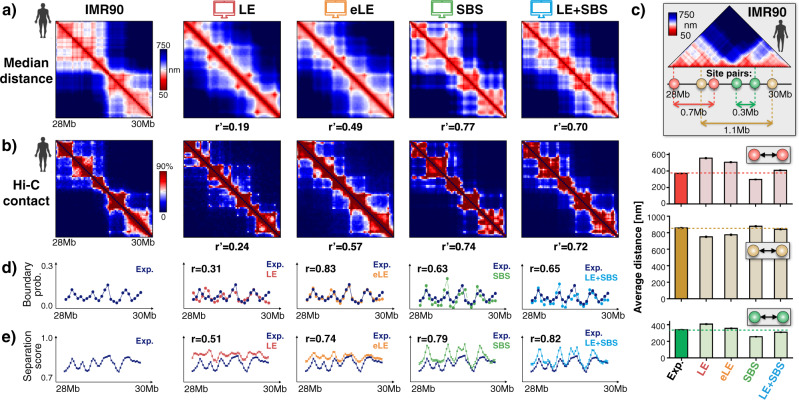
Both loop-extrusion and phase-separation based models recapitulate bulk Hi-C and average microscopy data. **a** Microscopy median distance^6^ and **b** bulk Hi-C^15^ data are compared to the corresponding model results in the IMR90 locus. The different models have high genomic distance-corrected Pearson correlations (*r*', reported below their matrix) with the experiments. **c** The model derived average distances are reported for three specific pairs of sites: (i) separated by a strong TAD boundary (yellow pair); (ii) connected in loops within a TAD (green) and (iii) across a TAD boundary (red). **d** The average genomic boundary probability across single-molecules and **e** the separation score are also well recapitulated by the models (error bars indicate 95% confidence intervals). *n* = 1000 independent single-molecule conformations for each model. Source data are provided as a Source Data file.

In our IMR90 case study locus, we found that the models all capture the global patterns visible in the median distance matrix^6^ (Fig. 2a, Methods). To have a quantitative measure of similarity, we computed the genomic distance-corrected Pearson correlation coefficient, *r*', between model and experiment. The LE has *r*' = 0.19, while the eLE has *r*' = 0.49, highlighting a markedly improved similarity to the experiment. The data appear to be better captured by the SBS and by the LE+SBS models, as signaled by their higher correlations (*r*' = 0.77 and *r*' = 0.70, respectively). Analogous results are found by comparing the model contact matrices against Hi-C data^15^ (Fig. 2b, Methods): LE has *r*' = 0.24, eLE has *r*' = 0.57, while SBS and LE+SBS models comparatively better reproduce Hi-C contact patterns (*r*' = 0.74 and *r*' = 0.72, respectively). We also considered other measures of similarity, such as the simple Pearson correlation (Supplementary Table 1), which provided analogous results.

Next, we focused on the relative distances of specific, interesting pairs of sites in the IMR90 locus. Specifically, we considered: (i) a pair of sites (highlighted in green, see the scheme in Fig. 2c and Supplementary Table 2) located 0.3 Mb apart from each other within the same TAD, having a strong interaction; (ii) a pair of sites (red), located 0.7 Mb away in different sub-TADs, having a strong loop contact in the median distance matrix; and (iii) a pair of 1.1 Mb distant sites (yellow) from different TADs, separated by a strong TAD boundary. Albeit the genomic separation of the red pair is twice as large as the separation of the green, those pairs have a similar average spatial distance in the experiment, close to 400 nm, whereas the boundary separated yellow pair is >800 nm apart (Fig. 2c, Supplementary Table 2). We found that the different models all recapitulate those values (Fig. 2c, Supplementary Table 2) and, interestingly, the LE+SBS model is overall the closest to the experiment across those specific pairs of sites. Additionally, we checked that the distance distributions derived from the models are all similar to the corresponding microscopy distance distributions (Supplementary Fig. 6). We stress, however, that the specific values of those distances can depend on the minute details of the models, such as the shape of the interaction potential or loop-extruder size.

To assess how well distinct models capture different aspects of chromatin folding, we also computed the probability to find a TAD boundary at a given genomic location and the average separation score^6^ along the locus in single-molecule conformations (Fig. 2d, e, Methods). In the IMR90 case study locus, we found that the boundary probability and the boundary strength averaged over all genomic positions are similar across the different models and very close to the experimental values (Supplementary Fig. 7). The boundary probability as a function of the genomic coordinates of the locus, however, is better captured by the eLE model, which has the highest Pearson correlation with experimental data (*r* = 0.83, Fig. 2d), while the LE has the lowest correlation (*r* = 0.31). The SBS and LE+SBS models also provide a good fit to the data, having respectively *r* = 0.63 and *r* = 0.65. We also found that all the models provide a good overall description of the average separation score along the locus (Fig. 2e): the LE has the lowest correlation to the corresponding experimental data (*r* = 0.51), the eLE has *r* = 0.74, the SBS *r* = 0.79 and the LE+SBS model *r* = 0.82.

Our analysis of the HCT116 locus returned a very similar picture about the performance of the different models to describe average distance and Hi-C data (Supplementary Fig. 8a, b) as well as TAD boundary probabilities and separation scores (Supplementary Fig. 8c, d and Supplementary Fig. 9).

Taken together, our results show that both loop-extrusion and phase-separation based models are consistent with ensemble-averaged microscopy and bulk Hi-C data. While the LE model is only partially effective, the eLE, with its optimized anchor sites and molecule-to-molecule variability, performs even better in the description of those data and is the best to recapitulate the TAD boundary probability function. However, polymer models including globule phase-separation mechanisms (SBS and LE+SBS) have overall higher correlation values with average microscopy distance and Hi-C contact data, and better capture some local features of chromatin folding, such as the separation score.

### The models are overall consistent with chromatin structure at the single-molecule level

To quantitatively assess how effective are the different models in explaining chromatin structure at the single-molecule level, we took advantage of the mentioned super-resolution microscopy data^6^ and of the ensemble of polymer 3D conformations produced via our computer simulations.

**Fig. 3:**
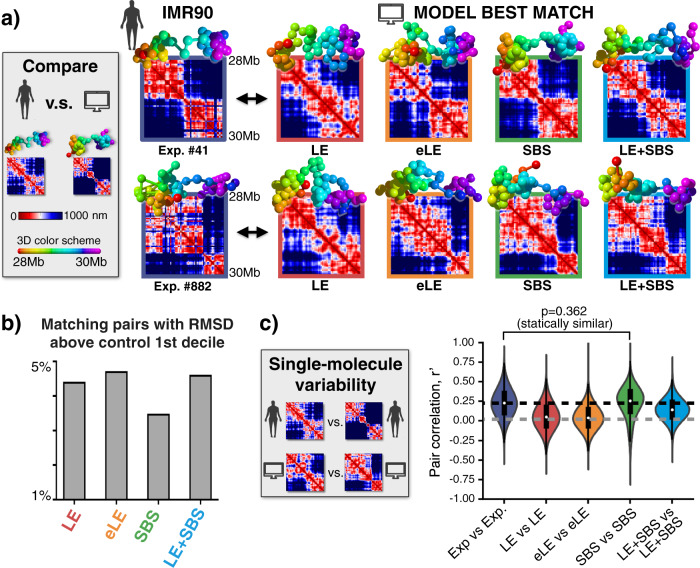
Single-cell chromatin conformations are well captured by the model 3D structures, especially by phase-separation based ones. **a** Microscopy single-cell chromatin structures of the IMR90 locus^6^ (left) are associated to their best matching single-molecule conformation in each model via the minimum RMSD criterion. Here two examples of best match are shown for each model type. **b** Less than 5% of the best-matching experiment-model pairs have an RMSD above the 1st decile of the control distribution. **c** The variability of microscopy single-molecule structures is measured by the distribution of *r*' correlations between pairs of distance matrices and is compared to the variability of in-silico structures. The *r*' distribution of the SBS model is statistically indistinguishable from the experimental one (two-sided Mann–Whitney test *p*-value = 0.362). The boxplots represent the median, interquartile ranges, whiskers within 1.5 times the interquartile range. *n* = 1000 independent single-molecule conformations for each model. Source data are provided as a Source Data file.

First, we checked how well each model represents single-cell chromatin conformations by performing an all-against-all comparison of single-molecule imaged and model 3D structures. We used a method^35,71^ whereby each 3D conformation from microscopy data is univocally associated to a corresponding model structure (for each considered type of model) by searching for the least root mean square deviation (RMSD) of their coordinates (Methods). Figure 3a and Supplementary Fig. 12a (in IMR90 and HCT116, respectively) show a few examples of those experiment-model best-matching structures, highlighting that, at least visually, each model appears to capture the overall structural pattern of its corresponding experimental conformation. To test the statistical significance of the association, we compared the RMSD distribution of the best-matching experiment-model pairs against a simple control case where the RMSD distribution is computed between random pairs of imaged structures. We verified that for each of the considered polymer models the RMSD distribution of the best-matching pairs is statistically different from the control in both the IMR90 and HCT116 loci (Fig. 10a,b two-sided Mann–Whitney test *p*-value < 0.001). Specifically, in the IMR90 locus we found, consistently across the models, that <5% of the former distribution is above the first decile of the control (Fig. 3b) and, in particular, the SBS model performs slightly better than the others. The analysis of the models of the HCT116 locus returned similar results (Supplementary Fig. 10c). As an additional test, we also considered a more stringent control where the RMSD is computed only between pairs of imaged structures having overall similar distance matrices, i.e., with a corresponding genomic distance-corrected correlation >0.5 (i.e., with *r*' > 0.5, see below), and we found analogous results (Supplementary Fig. 11). Hence, the model conformations best matching the experimental structures have a statistically significant RMSD distribution and provide a non-trivial description of chromatin molecules in single cells (Fig. 3a, Supplementary Fig. 12a).

Next, we tested whether the structural variability of model 3D conformations reflects the one observed in single-cell microscopy experiments^6^. In the IMR90 locus, for example, the distribution of *r*' correlations between pairs of experimental single structure distance matrices has an average *r*' = 0.23 and a variance equal to 0.18 (Fig. 3c), showing that while the imaged structures are broadly varying they have also a significant degree of similarity^6,35^. For each model, we computed the corresponding distribution of *r*' correlations between all model single-molecule distance matrices and we compared it with the experimental one (Fig. 3c and Methods). Interestingly, the *r*' distributions of the different models have all a shape similar to the experiment and a similar variance, yet they have different average values (Fig. 3c). The LE and eLE model average *r*' (*r*' = 0.06 and *r*' = 0.04, respectively) is significantly lower than the experimental value, showing that their single-molecule structures have a lower degree of similarity with each other than single-cell imaged chromatin conformations. The LE+SBS model has an average *r*' = 0.14, while the SBS model has *r*' = 0.23, which is equal to the microscopy value (Fig. 3c). In fact, the *r*' distribution of the SBS model is statistically indistinguishable from the experimental distribution (two-sided Mann–Whitney test *p*-value = 0.362), while the other models are statistically different (*p* < 0.001). Additionally, we verified that analogous results are found if the experiment-experiment *r*' distribution is compared to the distribution of *r*' correlations between experiment and model single-molecule distance matrices (Supplementary Fig. 13a). The analysis of the HCT116 locus returns a very similar scenario (Supplementary Figs. 12b, 13b). We also checked that our results are not affected by a different choice of the correlation parameter, hence confirming their statistical robustness (Supplementary Fig. 14). We stress, nevertheless, that those correlation measures can depend on the minute details employed to construct the models and the agreement with the experiment could be further improved.

Finally, as an additional test, we performed calculations of shape and volume factors of the single molecule structures predicted by our chromatin models, which we compared against single-cell imaging data^6^. To this aim, we computed for each imaged and model single molecule its corresponding inertia tensor, whose eigenvalues (i.e., the system principal momenta of inertia) are related to the semi-axes (named below *a*, *b*, *c*) of a triaxial ellipsoid enclosing the considered conformation (**Methods**). As a control, we considered a globule-like homopolymer model having the same number of beads and the same average gyration radius (that is, same linear size) as the microscopy images of the studied loci. In the IMR90 case, we found, interestingly, that the conformations predicted by the different models have a prolate shape that is consistent with imaging data (i.e., *a* = 0.8 µm > *b* ≈ *c* = 0.4 µm), whereas in the control the ellipsoid semi-axes are comparable to each other (i.e., *a* ≈ *b* ≈ *c*) (Supplementary Fig. 15a–c). Then, by using the ellipsoid semi-axes, we also computed for each type of model the distribution of the single-molecule volumes, which we found to be comparable with microscopy data (Supplementary Fig. 15d, average value 0.5 µm^3^). We performed all the above calculations in the HCT116 locus and found analogous results (Supplementary Fig. 16). As a further quantitative check, we calculated additional standard shape factors, such as the asphericity ratios^72,73^, and found similar results on the prolate shape of single-molecule conformations (Supplementary Fig. 17). Additionally, we measured the degree of spatial compaction of the single-molecule structures (Methods) and found that the distributions of compaction levels are comparable between experiments^6^ and our different polymer models (Supplementary Fig. 18a, b), and notably they have an overall profile similar to the independent relative signal distribution of DAPI nuclear intensity classes discovered in recent 3D-SIM experiments^62^ (Supplementary Fig. 18c).

In summary, consistent with our previous results on bulk data, our single-molecule analyses support the view that the different polymer models all provide a non-trivial description of single-cell chromatin conformations. While both loop-extrusion and phase-separation based models capture the main features of chromatin single-molecules, in the studied loci we find that the latter models better reflect the microscopy observed single-molecule globular structure and variability. In particular, our analysis shows that chromatin structure variability across single cells results from two main distinct, yet concurrent sources: on the one hand from the intrinsic degeneracy of folding that we find in all the considered models, and on the other hand from the differences of anchoring points (or, analogously, binding sites) in single-molecules, representing the epigenetic heterogeneity of single cells.

### The models well reproduce microscopy triple contact data

**Fig. 4:**
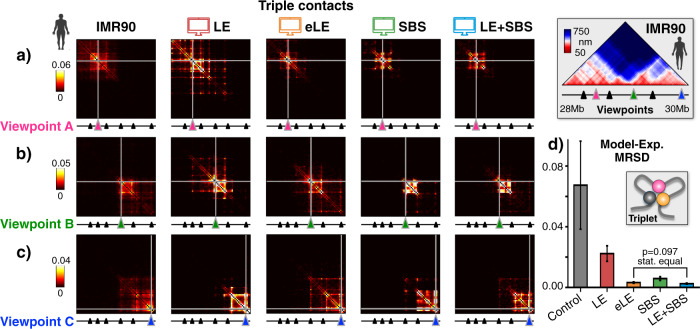
Triple contact data are well described by the models, especially by the eLE and the LE+SBS. Triple contact probability maps are shown in microscopy data^6^ (left) and in the models from three different viewpoints **a**, **b**, **c**, more viewpoints in Supplementary Fig. 19. **d** The mean relative squared difference (MRSD) between imaging and model triplet contact maps is the lowest in the LE + SBS model, which is statistically equivalent to the eLE model (two-sided Welch’s *t*-test *p* = 0.097). The control is made of randomly folded self-avoiding polymer chains with same number of beads and size than the experimental structures. Error bars represent SEM. *n* = 1000 independent single-molecule conformations for each model. Source data are provided as a Source Data file.

To assess how well the different models capture higher-order contacts, we investigated their predicted average triplet contact probability matrix, which we compared to microscopy data^6^ (**Methods**). We focused on triplets formed by six different genomic viewpoints roughly equally spaced along the IMR90 locus that correspond to some main TAD boundaries and loops of the pairwise median distance matrix (Fig. 4 and Supplementary Fig. 19). In our analysis, by definition, a triplet is formed when three genomic sites have all their pairwise distances below a threshold value. The triplet probability depends on such a threshold, but we checked that it is proportionally conserved if the threshold is varied around 150 nm, a typical value used in microscopy^6^, in a range from 100 to 200 nm (Methods).

Microscopy data reveal that triplets are typically compartmentalized in the studied loci and restricted to the TAD encompassing each of the selected viewpoints (Fig. 4a–c and Supplementary Fig. 19), showing that TADs tend to create local environments where also multiple contacts become enriched. The different polymer models do capture experimental triplet patterns across all the considered viewpoints. To quantitatively assess the similarity between experiment and model-predicted triplets, we computed the mean relative squared difference (MRSD) between the corresponding entries of the two matrices over the studied viewpoints (Fig. 4d, Methods). To set a reference, we also considered the triplets formed in a random control made of self-avoiding-walk (SAW) polymer chains having the same number of beads and gyration radius (i.e., linear size) as the microscopy images of the locus (Methods). Our analysis shows that the LE model has an MRSD with the experiment that is one third of the random control value, yet it has the largest discrepancy with microscopy data compared to the other considered models, whose MRSD is at least one order of magnitude smaller than the control. Interestingly, the LE+SBS model has the lowest distance from the experiment and its MRSD is statistically different from both the LE, the SBS and control case (Fig. 4d, two-sided Welch’s *t*-test *p* < 0.001), whereas it is statistically equal to the eLE MRSD (two-sided Welch’s *t*-test *p* = 0.097).

Taken together, our results show that both loop-extrusion and phase-separation mechanisms can explain higher-order contacts. However, a model combining both mechanisms (LE+SBS) has the least discrepancy with microscopy triplet data and overall provides an excellent description of all the different experimental datasets considered, supporting the view that loop-extrusion and phase-separation can coexist in single-molecules in establishing chromatin architecture.

## Discussion

To investigate the physical mechanisms that shape chromatin 3D large scale organization, we explored via Molecular Dynamics simulations two classes of polymer models where folding is based on two distinct physical processes: DNA loop-extrusion and polymer phase-separation, recapitulated respectively by the LE and by the SBS models (Fig. 1). We assessed how they perform relative to each other in capturing chromatin bulk Hi-C contact^15,63^ and independent single-molecule microscopy data^6^ in human IMR90 and HCT116 cells, and we exploited such data to establish whether those mechanisms compete or coexist in single cells.

We implemented, first, a simple loop-extrusion (LE) model^20^ of those loci (Fig. 1b) and found that it performs well to fit average microscopy distance and bulk Hi-C data (e.g., respectively, *r*' = 0.19 and *r*' = 0.24 in IMR90) considering the basic ingredients that inform the model. Next, we introduced an extended version of the LE (named eLE, Fig. 1c), where the genomic locations of the extruding motor anchor sites are optimized, independently of CTCF peaks, to explain average distance and contact data even better (respectively, *r*' = 0.49 and *r*' = 0.57). Beyond pairwise interactions, the eLE model also better recapitulates higher-order contacts in single molecules. Interestingly, most forward and reverse anchor sites of the optimized eLE model coincide with CTCF forward and reverse sites, yet not all, and conversely around 50% of all CTCF sites are found to be redundant (i.e., not required to better explain contact data) in the optimized model (Fig. 1c), hinting that CTCF sites are not all equal in the genome and can act in combination with other signals in anchoring loop-extruding motors^69,70^. Additionally, to mimic epigenetic differences among single cells, each of the eLE anchor sites has a specific probability to be present in a model single molecule^29^. The probability values returned by the optimization search range from 50% to 100%, consistent with current estimates of cell epigenetic heterogeneity^68^.

We also considered the SBS model of the studied loci (Fig. 1d), i.e., a model where the interaction between cognate binding sites on the polymer chain and their associated binding molecules drives a phase-separation of the chain in distinct globules^35^. For completeness, we checked that a model with direct interactions between binding sites (rather than mediated by diffusing binders) has behaviors analogous to the SBS. Finally, we introduced a model combining the molecular elements of the eLE and of the SBS (the LE+SBS model) where in a single molecule both the LE and SBS mechanisms act simultaneously (Fig. 1e). We find that the SBS and LE+SBS models explain well bulk Hi-C (e.g., respectively, *r*' = 0.74 and *r*' = 0.72 in IMR90), distance (*r*' = 0.77 and *r*' = 0.70) and single-molecule microscopy data, and reflect the experimentally reported chromatin segregation in globules and its cell-to-cell structural variability more accurately than the LE or eLE models (Fig. 3).

Importantly, a further optimization of the model fine details, such as the employed specific interaction potentials (shape, depth, distance of the potential minimum, etc.) or the specific nature of the modeled DNA extruding motors (size, speed, directionality, etc.), can on one hand improve even more the model agreement with experiments and on the other hand provide additional mechanistic information. Also, the LE and SBS models could be trained in a single step, which can only improve our results. Nevertheless, the models here investigated perform well considering their simplicity (Figs. 2–4). In particular, the LE+SBS model returns an overall excellent description of the different datasets and the least discrepancy with microscopy triplet data, showing that loop-extrusion and phase-separation can coexist in shaping loops, TADs and the complex 3D architecture of the studied loci. Our analyses also illustrate that the experimentally observed structural variability of chromatin in single-cells is consistent with two main co-existing sources of noise, i.e., the heterogeneity of single-cell epigenetics and, interestingly, an intrinsic conformational degeneracy, as chromatin can dynamically fold in many different conformations rather than in a single naïve structure as usual proteins.

Bulk Hi-C^63,74^ and microscopy^6^ experiments have shown that depletion of cohesin causes loss of TADs at the population level. Those results are consistent with the loop extrusion model as they remove the molecular factors which extrude loops. Analogously, they are consistent with the SBS and phase separation models because, by removing chromatin architectural factors, they reduce interactions and dilute contacts between cognate sites, resulting in random coil conformations where average patterns are erased. Indeed, in cohesin depleted cells, the single molecule structures reported by multiplexed microscopy^6^ were shown to be consistent with a reversal of phase-separated globular structures into more randomly folded states that abolish population-averaged domains^35^. Other perturbations, such as depletion of CTCF or of cohesin loader/unloader factors can be analogously explained. While the observed loss of TADs and structure upon cohesin (or CTCF) removal can be explained with both loop extrusion and polymer phase separation models, the debate is still open on the specific role of different chromatin factors. Recent studies on CTCF/cohesin depletion have shown that loop extrusion is not essential for establishing enhancer-promoter interactions (see, e.g.,^75–78^) or building functional compartments^62^, highlighting that chromatin contacts can rely on different factors, which is consistent with our results.

While other folding mechanisms, beyond loop extrusion and phase separation, are likely to contribute to the organization of the genome (such as heterochromatin adsorption to the lamina), one can speculate on why different molecular processes could cooperate in determining chromatin folding. Beyond ensuring redundancy in regulation, they appear to be more effective in implementing complementary tasks. For instance, loop-extrusion is particularly suited to establish TAD borders and pointwise strong loop interactions, whereas globule phase separation can better act to segregate different regions and to form more stable (i.e., with lower variability) and hence more reproducible regulatory structures. Additionally, while loop-extrusion requires energy consumption, phase transitions are sustained by the thermal bath, and they are robust and reversible processes as the system only needs, e.g., to set an above threshold concentration (or affinity) of binders, with no need of fine tuning their number (or strength).

## Methods

### The studied loci

In this paper we studied two 2 Mb wide loci in human IMR90 and HCT116 cells, where published single-cell imaging^6^ and independent in situ Hi-C data^15,63^ are available. The hg38 coordinates of the IMR90 locus are chr21:28000000–30000000, those of the locus in HCT116 cells are chr21:34600000–37100000. We used 5 kb resolution Hi-C data, KR normalized, re-binned by summation at 30 kb to match the resolution of multiplexed FISH data.

### Polymer models and simulation details

To investigate how loop-extrusion and polymer phase-separation models perform relative to each other in capturing chromatin folding in single-molecules, we implemented different versions of those models that we tested against independent multiplexed FISH data^6^ (Fig. 1, Supplementary Fig. 1). In each of the considered models, chromatin is represented as a polymer chain made of beads having a finite diameter, *σ*. All the model interaction potentials are taken from classical studies of polymer physics simulations^79^. Specifically, consecutive beads on the chain are connected via finitely extensible non-linear FENE springs with standard parameters^79^ (i.e., maximum bond length = 1.6*σ* and energy strength = 30*K*_B_*T*) and their overlap is prevented by a short-range steric interaction, implemented by a repulsive Weeks-Chandler-Anderson (WCA) potential^29,79^. Each polymer bead is subject to a Langevin dynamics, which is numerically integrated via the Velocity-Verlet algorithm by using the LAMMPS^80^ and HOOMD^81^ Molecular Dynamics (MD) software. In dimensionless units, the particles have all same diameter *σ* = 1, same mass *m* = 1, and a standard friction coefficient ζ = 0.5^79^. The polymer system is confined within a simulation cubic box with periodic boundary conditions. The initial states of our MD simulations are independent SAW conformations, prepared as detailed in^24,79^. Then, the main dynamics of the models is simulated (see below) and the polymer system evolves up to 10^8^ MD time iteration steps when stationarity is fully reached. For each type of model, we produced via massive parallel MD simulations an ensemble of 10^3^ single-molecule conformations in the steady state.

First, we considered a simple loop-extrusion (LE) model of the studied loci as described in^20^ (Fig. 1b). In brief, loop-extruding motors are modeled as additional harmonic springs on the chain, which extrude loops by translocating along the polymer. Their number is fixed, they cannot pass through each other and their translocation halts when they encounter another motor or anchor points with opposite orientation, or they stochastically dissociate from the chain. To simulate the extrusion process, at every *T*_ex_ time iterations steps, the simulation is updated by moving the spring from the bead pair (*i*, *j*) to (*i*−1, *j*+1). We set the LE spring energy constant equal to 10*K*_B_*T* and its rest length to 1.1*σ*, which are both values consistent with the literature range^19,20,29^. The time interval *T*_ex_ is equal to 500 timesteps, yet its specific value does not impact on the simulation results^19^. Positions and orientations of the anchor sites are determined by a standard motif finding analysis (using the FIMO tool within the MEME Suite software^66^) based on the peaks of available CTCF ChIP-seq data from ENCODE^67^ (ENCODE accession: ENCFF463FGL in HCT116; in IMR90 CTCF tracks are generated by ENCODE and downloaded from the UCSC website as in^6^). Each anchor site has a given probability, named strength^20^, to stall an LE motor at its position. Additionally, each anchor site is present in a given single polymer molecule with a given probability. In the LE model the strength and the presence probabilities of all anchor sites are both set equal to 100% in our simulations, i.e., the anchor points stall any LE motor at their position and they are fixed and equal across all the single-molecule conformations. We also checked that our results are overall unchanged if the anchor strengths are varied in a broad range, e.g., from 60% to 100%, or determined, for instance, via a logistic mapping transformation as in^20^.

To highlight the potential of the LE model and to dissect the roles of its ingredients beyond its minimal version, we developed a more refined, extended LE (eLE) model, which takes into account biological evidence (e.g., anchor sites changing from cell to cell) to fit even better the data. First, the polymer chain of the eLE model is subject to a generic self-attraction potential^19^ produced by unspecific bridging molecules that help better forming TADs and globules as seen in Hi-C and microscopy experiments. We set the binding energy affinity of such potential to 0.7*K*_B_*T*, yet we checked that different values, e.g., from 0.5 to 1*K*_B_*T*, return all similar results. Next, to mimic epigenetic differences across single cells, each anchor site of the eLE model has a specific, finite probability to be present in a single polymer molecule^29^ (Fig. 1c, Supplementary Fig. 1b). To best reproduce population-averaged Hi-C and microscopy distance data, we systematically searched for the optimal single-molecule presence probability and genomic locations of those anchor sites, independently of CTCF tracks. Specifically, by running extensive MD simulations, we explored up to 2x10^2^ distinct anchor site configurations in the studied loci to find the minimal set that best reproduces the average contact patterns. We sampled in our optimization search a wide spectrum of presence probabilities, spanning from 20% to 100%. The values returned by the procedure range roughly from 50% to 100%, which are overall consistent with current estimates of cell epigenetic heterogeneity^68^. In the studied loci, while interestingly the optimized eLE sites are all CTCF sites, we found that not all LE (FIMO) CTCF sites are retained in the model after the optimization as a fraction of them (roughly 50%) is redundant in the eLE (i.e., they are not required to explain contact data). For example, in our IMR90 main case, there are 16 CTCF FIMO sites, of which 11 are reverse- and 5 forward-oriented, that are used as anchor sites of the LE model (Supplementary Fig. 2a). In the eLE model, we found instead 12 anchor sites, of which 6 are reverse and 6 forward oriented (Supplementary Fig. 2b). Specifically, the 6 reverse-oriented sites of the optimized model (eLE) all match FIMO reverse-oriented sites, but 5 out the 11 FIMO reverse-oriented sites (i.e., roughly 45%) are found to be redundant in the optimized model. Analogously, 4 out the 6 forward-oriented sites of the optimized model match forward-oriented FIMO sites, whereas 2 forward eLE sites are found to match instead reverse-oriented FIMO sites and are unique to the optimized model; finally, 1 forward-oriented FIMO site is redundant in the eLE (Supplementary Fig. 2a, b). Additionally, while the eLE anchor sites are all characterized by CTCF/cohesin signatures, they also have overlaps with different chromatin marks such as H3K27ac, H3K4me1, H3K27me3 and Pol-II (Supplementary Fig. 2c), supporting the view that in the genome not all CTCF sites are equivalent, as reported in recent experiments^69,70^, and that they could be combined with other signals in determining loop sites. Similar results are found in the HCT116 cell locus, where to explain microscopy data the eLE model requires only a subset of the FIMO CTCF sites (19 out 47, roughly 40%) which are also located in correspondence of other histone and transcription marks (Supplementary Fig. 3). We stress, nevertheless, that those estimates are limited to the considered genomic loci and require genome-wide statistics to be robust. Finally, as in the LE model, the strength of the eLE anchor points is set to 100%, yet different values, e.g., in the range down 60%, provide similar results. The extrusion dynamics of the eLE model is the same as the above-described LE model. A crucial parameter controlling the system dynamics is the ratio between the extrusion velocity and the unbinding rate, named the extruder processivity^29,82^. In our simulations, we performed a systematic sweep of processivity values within the literature range^20,29^, e.g., from 80 kb up to 750 kb, and found that 700 kb produces the best agreement with average distance and contact data in the studied loci. Similarly, we also varied the number of loop-extruders, *N*_le_, in the range of previous studies^19^, e.g., from 5 to 20, and found that *N*_le_ = 10 provides the best results.

Next, we considered the SBS model of the studied loci^35^, where a chromatin region is represented as a Self-Avoiding Walk (SAW) chain of beads, along which different specific, as well as unspecific, types of binding sites (visually represented by different colors) are located for cognate molecular binders (Fig. 1d, Supplementary Fig. 1c). The binders are diffusing Brownian particles that move under the Langevin equation and they can bridge their cognate sites on the polymer via specific attractive interactions, hence driving a phase-separation of the chain in distinct globules. As the number of binders or their energy affinity grows above a given threshold, the model undergoes a thermodynamics phase transition from a coil, i.e., open randomly folded, to a phase-separated globule state, in which the specific interactions between cognate sites guide the self-assembly of the chain into spatially segregated globules. As stated by polymer physics^65^, the equilibrium conformations of the model fall in those two main folding classes that correspond to the system thermodynamics phases. The genomic locations of the binding sites of the SBS model are inferred by a machine learning procedure based on our previously published PRISMR method^28,35^, which takes as in input only bulk Hi-C data without a-priori epigenetic information or additional parameters. In our studied loci, the procedure returns seven distinct types of binding sites in IMR90 and four in HCT116, each type associated to a specific combination of chromatin architectural factors (included but not limited to, e.g., CTCF and cohesin^62^), as fully detailed in^35^. So, the SBS inferred site types of our models are different because, consistently, the distributions of chromatin marks of the two loci are different. A thermodynamic ensemble of single-molecule conformations of those loci is derived by running extensive MD simulations of the optimal polymer models inferred by PRISMR. Full details of the MD implementation of the SBS model can be found in^24,28^. We also explored in our model a scenario where chromatin is folded in different states in different single-cells^24,28^. To this aim, we considered a population mixture of polymer conformations, each spontaneously folded in one of the conformational classes predicted by polymer physics (i.e., the coil or the globule phase-separated state) and found, for example, that an ensemble of 3D structures, composed 90% of single-molecule conformations in the globule and 10% in the coil state, best explains all the different experimental datasets considered. In all our analyses, we set the SBS simulation parameters, such as binder concentrations and affinities, as in^35^. Interestingly, by taking, for example, a binder concentration in the range 0.05–0.5 µmol/l (values reported in^35^), corresponding to 30–300 binders/µm^3^, the predicted number of binders required to fold our considered genomic loci (whose volume estimates are close to 0.5 µm^3^, see Supplementary Figs. 15, 16 and below) ranges from tens to several hundreds, which are values consistent with typical transcription factor concentrations. Similarly, assuming a typical nuclear volume of 500 µm^3^, the number of binders to condensate the entire genome is expected to be larger by three orders of magnitude (e.g., in the range 15,000–150,000). Those predicted numbers represent an additional possibility to test our polymer models of chromatin, for instance by SMLM or STED experiments that are enabling to visualize chromosome 3D structure in individual nuclei with nanometer-scale precision^83,84^.

Additionally, we also implemented a variant of the SBS model where the physical interactions between cognate DNA sites are direct rather than mediated by binders and our conclusions remain unchanged as expected from Statistical Mechanics^65^. For instance, in the IMR90 locus, we checked that such a variant of the model returns bulk interaction patterns similar to the experiments and to those of the SBS (Supplementary Fig. 4). In fact, the model with direct interactions has high genomic-distance corrected Pearson correlations, *r*' (see below for definition), with the imaged median distance^6^ and average Hi-C^15^ contact maps of the locus (*r*' = 0.75 and *r*' = 0.58, respectively) as much as high *r*' values with the corresponding maps of the SBS model (*r*' = 0.71 and *r*' = 0.65). All the simulation parameters of the SBS with direct interactions are set as in^35^.

Finally, to investigate whether DNA loop-extrusion and polymer phase-separation could coexist in shaping the 3D architecture of the studied loci, we introduced a model combining the above-described eLE and SBS models (named the LE+SBS model), in which both mechanisms act simultaneously in a single polymer molecule (Fig. 1e, Supplementary Fig. 1d). Specifically, in our implementation, the polymer is initially in a SAW state and the binders are randomly located within the simulation box. The binding sites of the LE+SBS chain are those inferred by PRISMR for the SBS models of the studied loci^35^. The polymer model also includes now the specific, optimal sites of the corresponding eLE model, which are present in a single polymer molecule with the probability derived for the eLE model and act as anchor points in the extrusion dynamics (see above). While the binders interact with the specific and unspecific binding sites, after *T*_ex_ timesteps the loop-extruding springs stochastically bind to (and unbind from) the polymer at a constant rate and extrude loops as discussed before. In the MD implementation of the model, the Langevin equation regulates the SBS dynamics of polymer beads and binders and it is integrated with a constant time step *Δt* = 0.01^29^, while the LE dynamics is advanced at regular *T*_ex_ = 500 timesteps (in *Δt* units) starting from the final spring positions of the previous LE MD step as detailed in^20^. The simulation parameters of the LE+SBS model, such as the binder concentrations and affinities, or the number of LE springs and their processivity, are those stated above for the SBS and eLE models of the studied loci. Hence, in our approach, the LE+SBS model is not trained de novo, as it relies on the parameters of eLE and SBS trained separately. We showed that such a model combining eLE and SBS (trained separately) performs well in explaining chromatin contact data (Figs. 2–4), so we expect that an alternative, unified model that combines the learning of the LE and SBS into a single step can only improve on that.

We used the standard MD conversions to map the dimensionless units of our MD simulations into physical values^35,73,85^. The length scale of each model, i.e., the bead diameter *σ*, is set by equating the medians of the model and microscopy^6^ gyration radius distributions as in^35^ (Supplementary Fig. 5). We found: *σ* = 36 nm (for the LE model), *σ* = 53nm (eLE), *σ* = 60 nm (SBS), *σ* = 61 nm (LE+SBS) in the IMR90 case; *σ* = 41 nm (eLE), *σ* = 45 nm (SBS), *σ* = 53 nm (LE+SBS) in HCT116. The plots of the experimental and model 3D conformations in Fig. 3a and Supplementary Fig. 12a are produced with the POV-RAY software (Persistence of Vision Pty. Ltd., 2004). A simple linear spline interpolation is performed in the rendering of both experimental and model 3D structures.

### Spatial distance matrices and correlations

For each of the studied polymer models, the single-molecule distance matrix is the square matrix of the Euclidean distances between all pairs of polymer beads of the considered single-molecule conformation. It is efficiently computed in Python by using built-in functions within the SciPy package. The median distance matrix is then the ensemble median of the single-molecule distance matrices. To quantitatively compare median distance maps from models and experiments, we used the genomic distance-corrected Pearson correlation coefficient, *r*', which accounts for genomic distance effects^28^. Specifically, *r*' is the Pearson correlation computed on distance (or contact) matrices whose diagonals are subtracted, in both models and experiments, by their average value at that genomic distance. The patterns of the microscopy median distance matrix^6^ are well captured by the different models, yet the SBS and LE+SBS have overall higher *r*' correlation values with the experiments (Fig. 2a and Supplementary Fig. 8a). We also considered other measures of similarity, such as the usual Pearson correlation, *r*, which returned similar results (Supplementary Table 1). Distance matrices are visually represented as 2-dimensional heatmaps with the seismic reversed color bar as in^6^. The color bar scale limit of the models is set equal to the experimental range to have a fair comparison. We also verified that a lower scale limit in the models (e.g., equal to zero) does not change our results, as done, for instance, for the SBS and LE+SBS models in Fig. 2a and Supplementary Fig. 8a. To deal with missing values in the data, we excluded from all our analyses imaged single-cell distance matrices having missing values for >80% of their entries and we also checked for outliers by removing in both experiments and models, as in^35^, the matrices with a low average *r*' (*r*' < 0.1) against the others. Finally, we investigated the relative distances of specific site pairs (red, yellow, green in Fig. 2c), which correspond to the following genomic coordinates (hg38) in IMR90: red: 28.06–28.72 Mb; yellow: 28.54–29.65 Mb; green: 29.08–29.38 Mb.

### Pairwise contact frequency maps

The average pairwise contact matrix of each of the considered polymer models is computed based on a standard method used in the literature^24,46^. Specifically, for each single-molecule conformation, we first derive the corresponding pairwise contact map, i.e., a symmetric square matrix whose entry (*i*, *j*) is equal to one if the polymer beads, *i* and *j*, are in contact, and zero otherwise. A contact between two any polymer beads is established if their relative spatial distance is less than a distance threshold *Aσ* (*A* is a dimensionless constant). Then, for each model, the average pairwise contact map is simply the ensemble average of the single-molecule contact matrices. We set, as in^35^, the constant scale factor *A* equal to 3.5 and 5, respectively, in the IMR90 and HCT116 models, yet we checked that changing those numbers, e.g., from three up to ten, only marginally affects our results. As before, the model contact matrices compare well against Hi-C data^15,63^, especially in the case of the eLE, the SBS and LE+SBS models, as signaled by their high *r*' correlation values (Fig. 2b, Supplementary Fig. 8b).

### Boundary probability and separation score

TAD boundary probabilities and strengths are computed, in both experiments and models, by using the definitions and algorithms published in^6^. In particular, for the experiments, as well as for the LE and eLE models, the algorithm parameters are set as^6^: gb = 1, valley = 1, su = 10, sl = 6. We also verified that upon changing those numbers, our general results, such as boundary locations and strengths, are overall preserved. For instance, in the case of the SBS and LE+SBS models we used: gb = 1, valley = 8, su = 4, sl = 4 in IMR90 and gb = 1, valley = 4, su = 5, sl = 5 in HCT116, as in^35^. The polymer models turned out to provide all a good description of the boundary features of the studied loci. For example, for each type of model, the boundary probability averaged over the genomic coordinates is comparable with the expected value from imaging data (Supplementary Figs. 7a, 9a, error bar is the standard error of the mean). Also, the boundary strength distributions of the models are all similar to the experiments (Supplementary Figs. 7b, 9b), as well as their corresponding average values (Supplementary Figs. 7c, 9c, error bar is the standard deviation divided by the square root of the number of boundaries). Finally, the comparison of the experimental and model derived genomic boundary probability functions also provides high Pearson correlation values (Fig. 2d and Supplementary Fig. 8c, a two-point running average is performed to better visualize those functions). We stress that no free parameters are available in all those calculations and comparisons.

Similarly, by using the definitions and methods reported in^6^, we computed the separation score as a function of the genomic coordinates along the studied loci (Fig. 2e and Supplementary Fig. 8d, error is 95% confidence interval). The different models well recapitulate the experimental functions, particularly the LE+SBS, which has the highest correlation to the data in both the IMR90 and HCT116 loci (*r* = 0.82 and *r* = 0.90, respectively). Again, no free parameters are available in those comparisons.

### All-against-all comparison of single-molecule imaged and model 3D structures via the minimum RMSD criterion

To check how well the different models represent the ensemble of single-molecule imaged conformations of the studied chromatin loci, we performed an-all-against-all comparison of microscopy and model 3D structures. To this aim, we used an accepted method^35,71^ that performs a rotational and translational alignment of two structures (e.g., experimental and model derived) by minimizing the root mean square deviation (RMSD) of their particle positions. Thus, by searching for the least RMSD, each conformation from microscopy data^6^ is univocally associated to a corresponding, best-matching structure of the models (for each type of model). Examples of experiment-model best-matching pairs are in Fig. 3a and Supplementary Fig. 12a, respectively for the IMR90 and HCT116 loci. To efficiently perform the structural comparison, we used the free available MDAnalysis Python library, which uses the fast QCP algorithm to compute the RMSD between two coordinate sets^86^. A standard z-score is applied on the experimental and model coordinates to have a fair comparison and missing values are linearly interpolated. To prove the association is statistically significant, we first tested the RMSD distribution of the experiment-model best matches against a loose control made of random pairs of imaged structures. For each of the considered polymer models, we checked that in both the IMR90 and HCT116 loci the two distributions are statistically different (Supplementary Fig. 10a, b, two-sided Mann–Whitney test *p*-value < 0.001) and well separated (Fig. 3b and Supplementary Fig. 10c, <5% and 15% of the best-matching pairs is above the first decile of the control, respectively, in IMR90 and HCT116). Next, to further test our association, we performed an additional, more stringent control where the RMSD is computed only between pairs of microscopy conformations having distance matrices with a corresponding genomic distance-corrected Pearson correlation value *r*' > 0.5 and found again analogous results (Supplementary Fig. 11). Specifically, in both loci, the RMSD distribution of the best-matching pairs of each model is statistically different from the control (two-sided Mann–Whitney test *p*-value < 0.001) with <25% of entries of the former falling above the first quartile of the latter. Finally, we also checked that the comparison against a control SAW model with the same number of particles and 3D size as the imaged conformations returns similar results, as shown in a previously published paper^38^.

### Structural variability of single-molecule conformations

To quantify the level of structural variability of single-molecule conformations, we computed the all-against-all distribution of *r*' correlations between pairs of single-molecule imaged^6^ and model distance matrices. In the IMR90 locus, the experiment-experiment *r*' distribution is broad (variance 0.18) and has a non-zero average value (*r*' = 0.23), signaling that the imaged single-cell conformations have a significant degree of structural similarity, yet they are broadly varying. The corresponding *r*' distributions from the models have all a similar shape and variance, but they return different average values: *r'* = 0.06, *r*' = 0.04, *r*' = 0.23, and *r*' = 0.14, respectively, in the case of LE, eLE, SBS, and LE+SBS (Fig. 3c). Similarly, in the HCT116 locus, the distribution of *r*' correlations between experimental single-cell distance matrices has an average *r*' = 0.27, while the corresponding average values from the models are: *r*' = 0.07, *r*' = 0.30, and *r*' = 0.23, resp., for the eLE, SBS, and LE+SBS (Supplementary Fig. 12b). We also compared, for each type of model, the experiment-experiment *r*' distributions against the distribution of *r*' correlations between experimental and model single-molecule distance matrices and found analogous results (Supplementary Fig. 13). Additionally, as a further check, we also transformed the *r*' correlation parameter through the Fisher z-transformation in all the comparisons between models and experiments made in the studied loci and found that such transformation does not affect our results, hence confirming their statistical robustness (Supplementary Fig. 14). We used the Python open-source Pandas tools to efficiently compute the *r*' distributions and the Python Seaborn data visualization library to produce the corresponding violin plots. Each violin plot is the combination of a standard boxplot (extending from the lower to upper quartile values of the data) and a kernel density estimation of the underlying distribution. To fairly manage missing values in the data, the *r*' pair correlation between single-molecule distance matrices is computed only between the entries with numerical values, excluding NA/null values in both input arrays. Also, to reduce the noise, we performed a standard Gaussian filter on single-cell distance matrices (standard deviation of the Gaussian kernel equal to 1). Statistical similarity between *r*' distributions is assessed by a two-sided Mann–Whitney test, computed only between independent pairs of distance matrices as in^35^. Finally, as also stated in the Main Text, the above correlation measures can depend on the finer details of the models, hence the agreement with the data could be further improved.

### Volume and shape factors of single-molecule conformations

To infer quantitative information about chromatin shape of the studied loci, we computed the inertia tensor, $I$, of each imaged^6^ and model single-molecule 3D structure, defined as: ${I}_{{jk}}=\mathop{\sum }\limits_{i=1}^{N}{m}_{i}({r}_{i}^{2}{\delta }_{{jk}}-{x}_{{ij}}{x}_{{ik}})$, where *N* is the number of beads of each single structure, *m*_i_ the mass of the *i-*th bead particle and *x*_ij_ its *j*-th spatial coordinate (*j*,*k* indices are equal to 1,2,3, respectively, for *x*,*y*,*z*). By diagonalizing $I$, we derived its three eigenvalues, i.e., the system principal moments of inertia ${I}_{a}$, ${I}_{b}$, ${I}_{c}$. Those moments are related to the semi-axes (named below *a*, *b*, *c*) of a triaxial ellipsoid enclosing each considered single-molecule conformation via the relations: ${I}_{a}={\frac{N}{5}}({b}^{2}+{c}^{2})$, ${I}_{b}={\frac{N}{5}}({a}^{2}+{c}^{2})$, ${I}_{c}={\frac{N}{5}}({a}^{2}+{b}^{2})$. To establish a control, we considered a globule-like homopolymer model having the same number of beads and the same average gyration radius (that is, same size) as the microscopy images of the studied loci. In our IMR90 main case, we found, interestingly, that the conformations predicted by the LE, eLE, SBS and LE+SBS models have all a prolate shape that is consistent with imaging data (i.e., *a* > *b* ≈ *c*), whereas in the control model the ellipsoid semi-axes turned out to be comparable to each other (i.e., *a* ≈ *b* ≈ *c*) (Supplementary Fig. 15a, b, boxplots extend from the lower to upper quartile values of the data, with mean highlighted in yellow). To further test such a prediction, we computed the flattening parameter defined as (*a-b*)/*a*, which is a measure of the degree of ellipticity of the ellipsoid shape: the flattening of the control homopolymer, as expected, is centered around 0, whereas our different models return all an average value close to 0.5 that is again consistent with imaging data (Supplementary Fig. 15c). Then, we computed for each imaged and model single-molecule 3D structure the volume of the corresponding bounding ellipsoid by using the relation *V* = (4/3)π*abc* (Supplementary Fig. 15d). We found that the single-molecule volume distributions across the LE, eLE, SBS and LE+SBS models are comparable with the experiments (average value 0.5 µm^3^, Supplementary Fig. 15d), consistent with the findings of our manuscript. Additionally, we performed all the above calculations in the other HCT116 cell locus and found analogous results (Supplementary Fig. 16).

As a further quantitative check, we calculated additional standard shape factors, such as the asphericity ratios of single-molecule conformations measured by the eigenvalues of their gyration tensor (see, e.g., refs. ^72,73^ for reference), and found similar results on the prolate shape of single-cell imaged and model 3D structures (Supplementary Fig. 17).

Finally, we exploited the above volume estimations to measure the degree of spatial compaction of model and real chromatin single-molecules of the studied loci. In particular, recent 3D-SIM experiments allowed to identify the patterns of local chromatin density in the cell nucleus, leading, for example, to the identification of seven DAPI intensity classes associated to local differences in DNA compaction^62^. Thus, to assess whether our models recapitulate the patterns of chromatin compaction found at the nuclear scale by those independent 3D-SIM data, we computed for each experimental^6^ and model 3D conformation the ratio <*V*>/*V* (where *V* is the ellipsoid volume enclosing the single-molecule structure and <*V*> its ensemble averaged value), which provides a normalized measure of the degree of chromatin spatial compaction. Then, as done in^62^, we divided the distributions of compaction levels (<*V*>/*V*) of our studied loci into seven, equally spaced classes and measured their relative abundance, i.e., the relative fractions of single-molecules within each class (Supplementary Fig. 18; outliers below first and last decile are removed in both experiments and models). We found a distribution of compaction levels consistent between imaging data^6^ and the different models of our studied loci (Supplementary Fig. 18a, b, statistical error is SEM; classes are ordered from lower to higher compaction) and, interestingly, also similar to the relative signal distribution of DAPI intensity classes reported by 3D-SIM (Supplementary Fig. 18c, taken from^62^). Overall, our analyses on volume and shape factors of single molecules further support our results and also highlight the general relevance of other microscopy methods (e.g., 3D-SIM^62^) through the predictions of our polymer models of chromatin.

### Triplet contact frequency maps

To investigate the higher-order structure of the studied loci, we computed from our models the predicted frequencies of triplet contacts that we compared against microscopy data^6^. Specifically, in our analysis we considered six different genomic viewpoints, which are roughly equally spaced along the IMR90 locus and proximal to the main TAD boundaries and loops of the distance matrix. Their coordinates are (hg38): 28.33–28.36 Mb, 28.51–28.54 Mb, 28.72–28.75 Mb, 29.08–29.11 Mb, 29.38–29.41 Mb, 29.83–29.86 Mb. For each of the studied viewpoints, *k*, we computed the corresponding average triplet contact probability matrix in both models and experiment (Fig. 4 and Supplementary Fig. 19), i.e., a symmetric square matrix whose entry (*i*, *j*) is the frequency of the triplet contact between the sites *i*, *j*, and the fixed viewpoint *k*. Those sites, by definition, form a triplet only if their relative pairwise spatial distances, i.e., *r*_ij_, *r*_ik_ and *r*_jk_, are all below a given threshold value^33^. In the experiment, for instance, we set such a threshold equal to the reference value^6^ 150 nm, but we also checked that different values within the range 100–200 nm proportionally preserve the measured triplet frequencies and contact patterns. In particular, a distance threshold of 180 nm in the models turned out to better recapitulate the experimental triplet frequencies. To measure the similarity between microscopy and model-predicted triplet contact maps, we computed, for each of the considered models, the mean relative squared difference (MRSD) between the corresponding entries of the two matrices, defined as the mean value of the ratios (*A*_ij_-*B*_ij_)^2^/*A*_ij_, where *A* is the experimental triplet matrix (for a fixed, considered viewpoint) and *B* the corresponding prediction of the model. The ratios are evaluated only for non-zero *A*_ij_ entries. In Fig. 4d we report for each model the MRSD averaged over the considered viewpoints (error bars are SEM). As a control, we computed the triplets in a SAW polymer chain with the same number of beads and gyration radius as the imaged conformations. While the random control returns the largest MRSD with the experiment, the LE+SBS has, interestingly, the lowest difference with the data and its value is statistically equal to the eLE MRSD (Fig. 4d, two-sided Welch’s *t*-test *p* = 0.097).

### Reporting summary

Further information on research design is available in the Nature Research Reporting Summary linked to this article.

## Supplementary information

Supplementary Information

Reporting Summary

## Data Availability

The data that support this study are available from the corresponding author upon reasonable request. Published Hi-C data^15,63^ used for analyses are available at the Gene Expression Omnibus (GEO) database with accession numbers GSE63525 and GSE104334. Published single-cell imaging data^6^ used in this study are available at https://github.com/BogdanBintu/ChromatinImaging. ChIP-seq data analyzed in this study were accessed via ENCODE^67^ (ENCODE accession for IMR90: ENCFF195CYT [https://www.encodeproject.org/experiments/ENCSR000EFJ/], ENCFF899APS [https://www.encodeproject.org/experiments/ENCSR002YRE/], ENCFF474OJM [https://www.encodeproject.org/experiments/ENCSR087PFU/], ENCFF752IXO [https://www.encodeproject.org/experiments/ENCSR831JSP/], ENCFF178QVF [https://www.encodeproject.org/experiments/ENCSR437ORF/], ENCFF741WIY [https://www.encodeproject.org/experiments/ENCSR431UUY/], ENCFF625BTD [https://www.encodeproject.org/experiments/ENCSR055ZZY/], ENCFF448ZOJ [https://www.encodeproject.org/experiments/ENCSR000EFK/], ENCFF732WRW [https://www.encodeproject.org/experiments/ENCSR000CTK/], ENCFF453XKM [https://www.encodeproject.org/experiments/ENCSR000EFI/]; for HCT116: ENCFF391AAM [https://www.encodeproject.org/experiments/ENCSR000BSB/], ENCFF899XEF [https://www.encodeproject.org/experiments/ENCSR661KMA/], ENCFF711MPL [https://www.encodeproject.org/experiments/ENCSR333OPW/], ENCFF931YSQ [https://www.encodeproject.org/experiments/ENCSR161MXP/], ENCFF137TPC [https://www.encodeproject.org/experiments/ENCSR091QXP/], ENCFF294LZM [https://www.encodeproject.org/experiments/ENCSR810BDB/], ENCFF832IOO [https://www.encodeproject.org/experiments/ENCSR179BUC/], ENCFF848IHI [https://www.encodeproject.org/experiments/ENCSR000EUU/], ENCFF870WXZ [https://www.encodeproject.org/experiments/ENCSR698RPL/], ENCFF463FGL [https://www.encodeproject.org/experiments/ENCSR240PRQ/]). Source data are provided with this paper.

## Source data

Source data

## Code availability

All the codes used in this work are based on standard, publicly available software packages, as detailed in the Methods section. The software used for Molecular Dynamics simulations is LAMMPS, version 30july2016, and HOOMD, version 2.9.x. 3D structures visualization was realized with the POV-Ray software, version 3.7. All the codes required to perform the simulations of the polymer models are provided at https://github.com/ehsanirani/PhaseSeparation-LoopExtrusion-MD^87^.
