# Supplementary material for: Loop-extrusion and polymer phase-separation can co-exist at the single-molecule level to shape chromatin folding
Source: Nat Commun. 2022 Jul 13;13:4070. doi: 10.1038/s41467-022-31856-6 (PMC9279381; doi:10.1038/s41467-022-31856-6)
Supplement: Supplementary file 1 — Supplementary Information [file 41467_2022_31856_MOESM1_ESM.pdf]

Supplementary Materials

for

Loop-extrusion and polymer phase-separation can co-exist at the single-molecule level to shape chromatin folding

Mattia Conte[1*], Ehsan Irani[2,3*], Andrea M. Chiariello[1*], Alex Abraham[1], Simona Bianco[2], Andrea Esposito[1], Mario Nicodemi[1,2,3,§]

[1]Dipartimento di Fisica, Università di Napoli *Federico II*, and INFN Napoli, Complesso Universitario di Monte Sant'Angelo, 80126 Naples, Italy.

[2]Berlin Institute for Medical Systems Biology, Max-Delbrück Centre (MDC) for Molecular Medicine, Berlin, Germany.

[3]Berlin Institute of Health (BIH), MDC-Berlin, Germany.

* These authors contributed equally.

§ Lead contact: mario.nicodemi@na.infn.it

SUPPLEMENTARY FIGURES

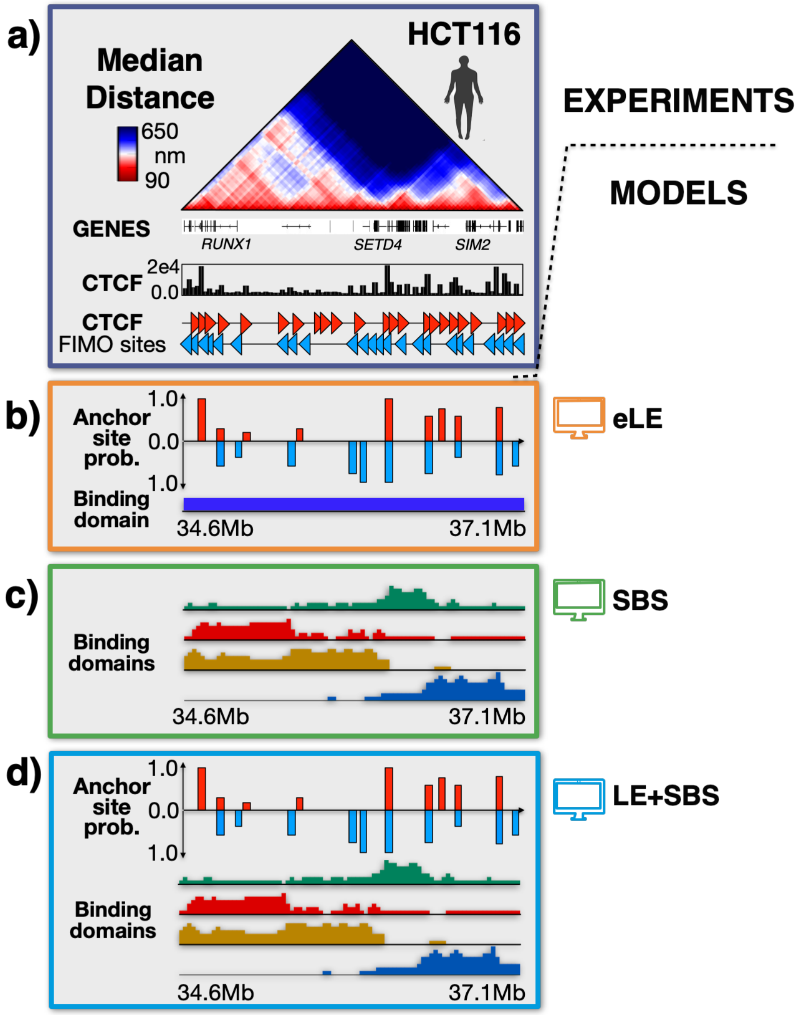

Supplementary Figure 1. Scheme of the investigated polymer models in HCT116.

a) Microscopy median distance[1] and ENCODE[2] CTCF data are shown for the studied 2.5Mb wide locus in human HCT116 cells. **b)** We considered an extended LE (eLE) model where the genomic locations of the anchor sites are optimized, independently of CTCF, to best reproduce Hi-C and average microscopy data. Also, to mimic single-cell epigenetic heterogeneity, those anchor sites have a specific, finite probability to be present in a model single molecule[3]. **c)** The Strings and Binders (SBS) model[4] of the locus has four distinct types of binding sites (represented by different colors), inferred by the PRISMR method and each correlated with different combinations of epigenetic factors including, but not limited to, CTCF and cohesin[4,5]. **d)** Scheme of the combined LE+SBS polymer model where both the eLE and SBS mechanisms act simultaneously in each single-molecule conformation.

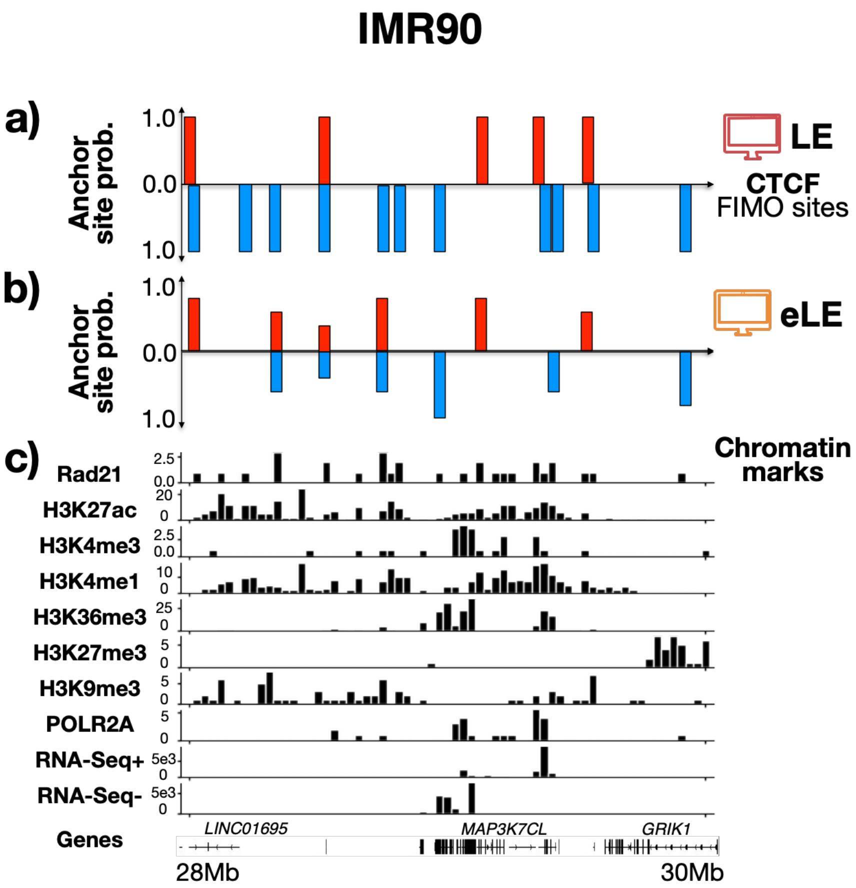

Supplementary Figure 2. Comparison between the LE (FIMO) CTCF and the eLE optimized anchor sites in the IMR90 cell locus.

a) Genomic locations and single-molecule presence probability of the CTCF FIMO anchor sites used in our LE model of the IMR90 cell locus (red bars represent forward- and blue bars reverse-oriented sites). **b)** Genomic locations and single-molecule presence probability of the anchor sites used in our eLE model. **c)** Chromatin marks from ENCODE[2] available in the studied locus (Chr21:28-30Mb). While the optimized eLE sites all match CTCF sites, a fraction (roughly 50%) of the LE FIMO CTCF sites are redundant in the optimized model, i.e., they are not required to explain contact data. That illustrates that CTCF sites are not all equal. In particular, different optimized eLE CTCF sites have different single molecule probabilities and, additionally, they typically correspond to peaks of other different chromatin marks, such as histone and Pol-II peaks.

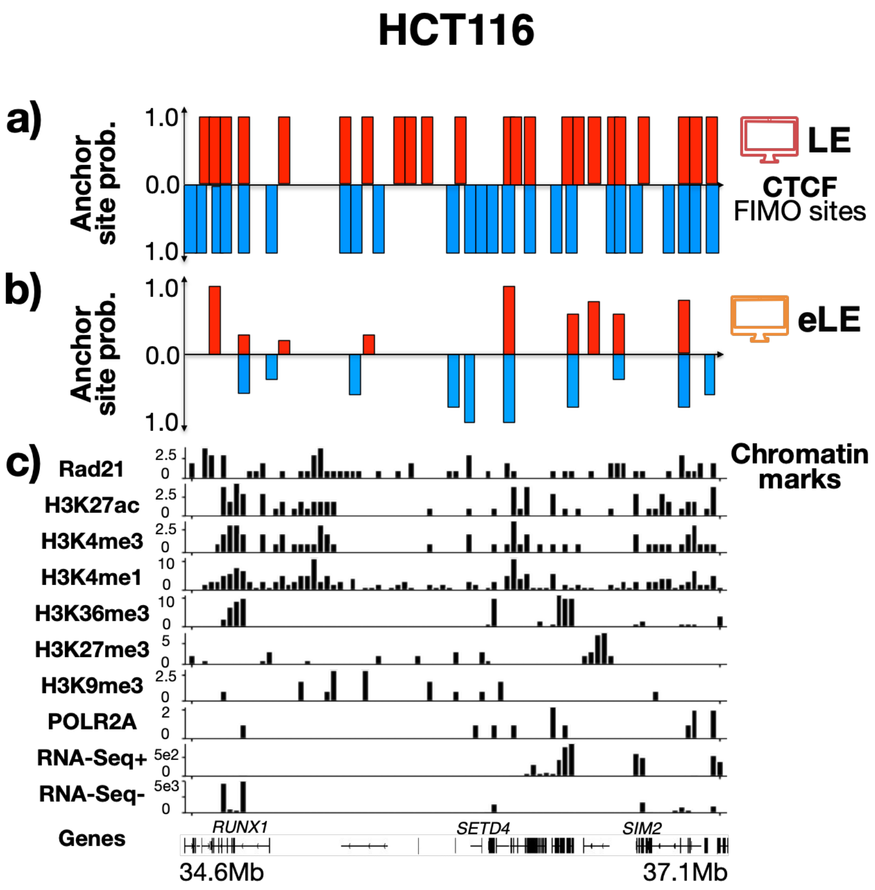

Supplementary Figure 3. Comparison between the LE (FIMO) CTCF and the eLE optimized anchor sites in the HCT116 cell locus.

a) Genomic locations and single-molecule presence probability of the CTCF FIMO anchor sites of the HCT116 cell locus (red bars represent forward- and blue bars reverse-oriented sites). **b)** Genomic locations and single-molecule presence probability of the anchor sites used in our eLE model. **c)** Chromatin marks from ENCODE[2] available in the studied locus (Chr21:34.6-37.1Mb). While the optimized eLE sites all match CTCF sites, a fraction (roughly 60%) of the LE FIMO CTCF sites are redundant in the optimized model. Also, the eLE CTCF sites have different single molecule probabilities and, additionally, they are typically located in correspondence of other histone and transcription marks.

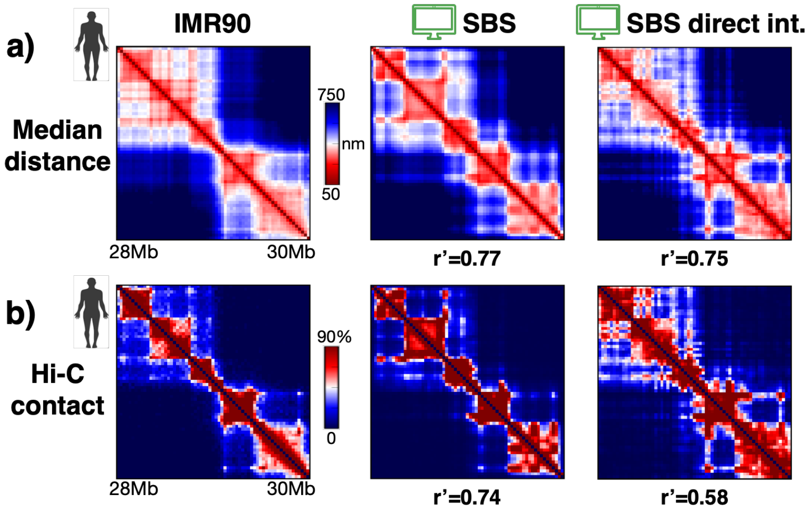

Supplementary Figure 4. A variant of the SBS model with direct interactions between DNA sites, rather than mediated by molecular binders, has behaviors analogous to the original SBS.

We explored a variant of the SBS model where cognate DNA sites have direct physical interactions, rather than mediated by diffusing binders. **a)** The median distance matrix[1] and **b)** the Hi-C contact map[6] of the IMR90 locus (left) are well recapitulated by both the SBS model (middle) and its variant with direct physical interactions (right), as quantified by their high genomic distance-corrected Pearson correlations (r') with the experiments. In particular, the model with direct interactions returns contact patterns similar to the SBS, as expected from Statistical Mechanics[7]. Source data are provided as a Source Data file.

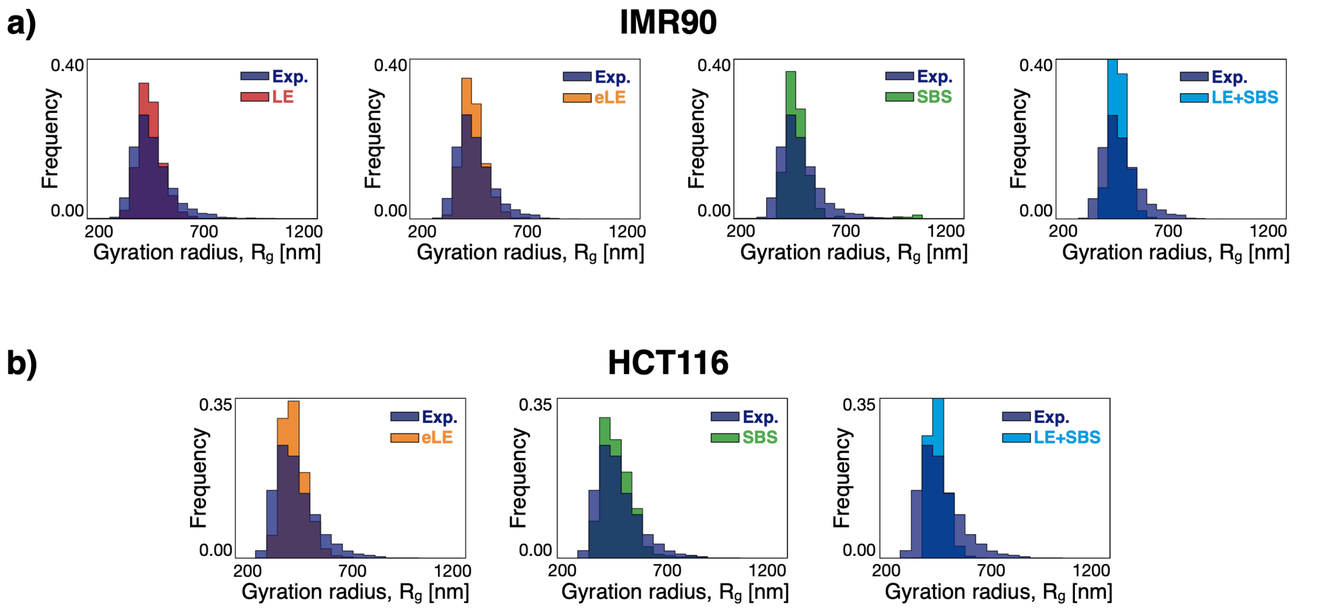

Supplementary Figure 5. Gyration radius distributions in imaging experiments and in the models of the studied loci.

Experimental[1] and model derived gyration radius distributions in the studied **a)** IMR90 and **b)** HCT116 loci. In all the considered cases, the model unit length scale is mapped into physical units by equating the median gyration radius to its corresponding experimental value.

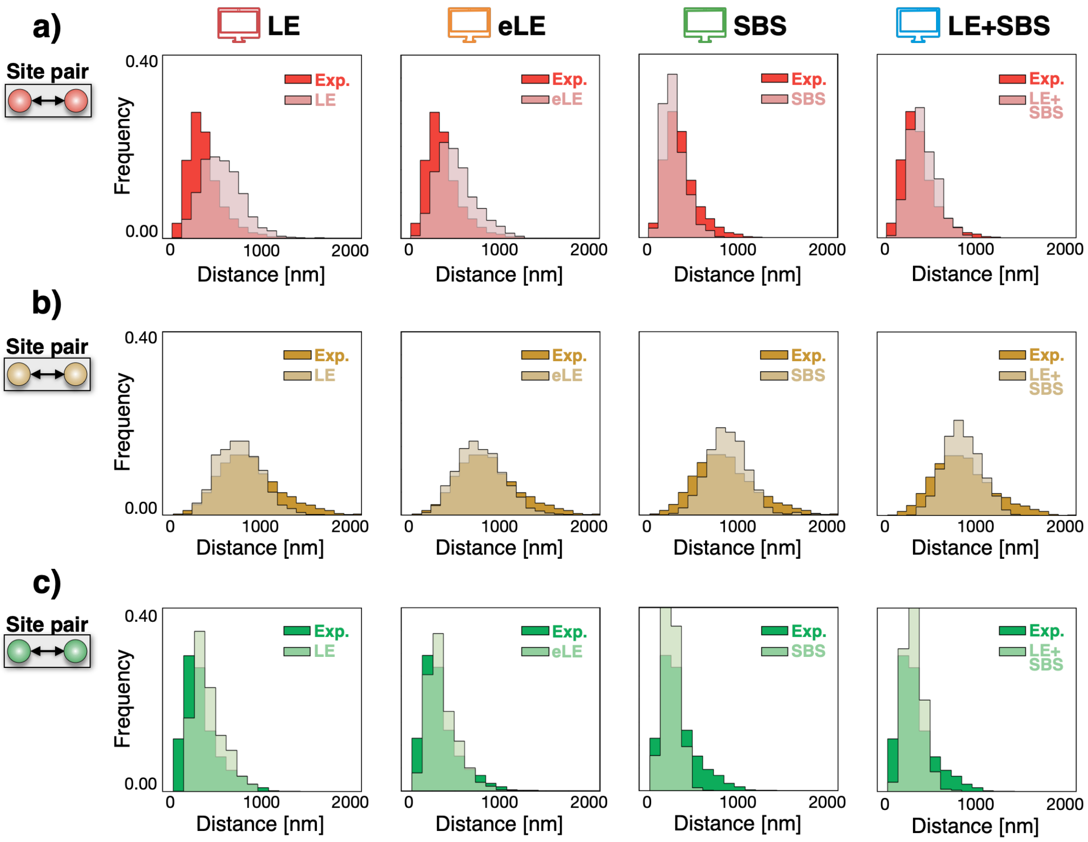

Supplementary Figure 6. Distance distributions of site pairs in the IMR90 locus.

We measured in both the experiment[1] and models the distance distributions of specific, interesting pairs of sites along the IMR90 locus. Distributions are shown for: **a)** a pair of sites (red), located 0.7Mb away in different sub-TADs and forming a strong loop contact in bulk data; **b**) a pair of sites (yellow) 1.1Mb apart from different TADs and separated by a strong TAD boundary in between; **c)** a pair (green) of 0.3Mb distant sites with a strong loop interaction in the same TAD. The distributions derived from the models are all similar to the corresponding imaged distributions, yet the specific values of those distances can depend on the minute details of the models.

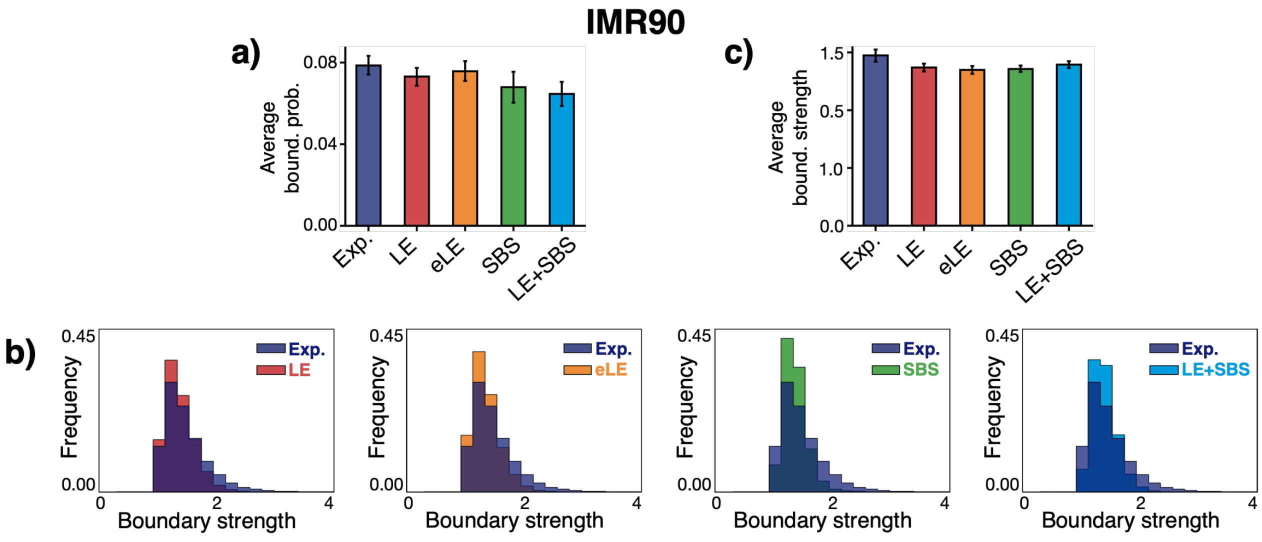

Supplementary Figure 7. The average boundary probability and strength are similar across the different models and close to the corresponding experimental values in IMR90.

a) TAD boundary probability averaged over all genomic positions in both experiment[1] and models in the IMR90 locus (error bars SEM). **b)** The distributions of the boundary strengths derived from the models are all similar to the experimental distribution, as well as **c)** their corresponding average values (error bars SEM). *n*=1000 independent single-molecule conformations for each model.

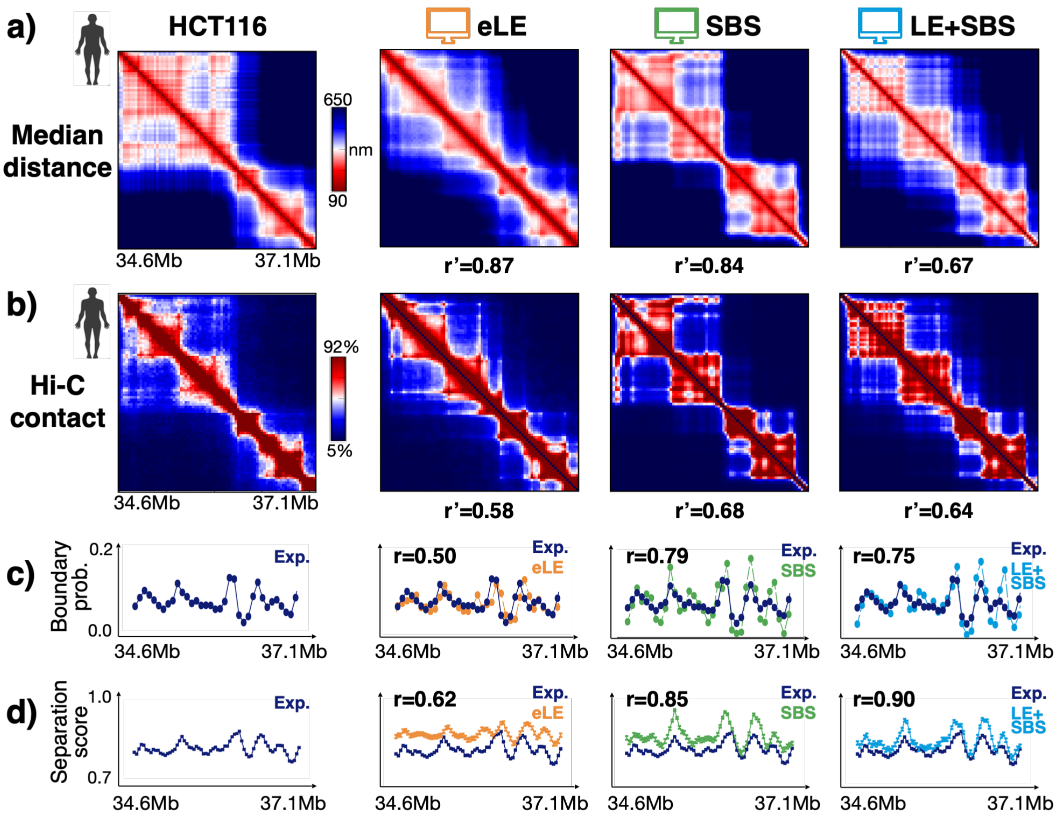

Supplementary Figure 8. Both loop-extrusion and phase-separation based models recapitulate bulk Hi-C and average microscopy data in the HCT116 locus.

a) Microscopy median distance[1] and **b)** bulk Hi-C[8] data (left) are compared to the corresponding model results in the HCT116 locus. The different models have all high genomic distance-corrected Pearson correlations (r', reported below their matrix) with the experiments. **c)** The average genomic boundary probability across single-molecules and **d)** the separation score are also well recapitulated by the models (error bars indicate 95% confidence intervals). *n*=1000 independent single-molecule conformations for each model. Source data are provided as a Source Data file.

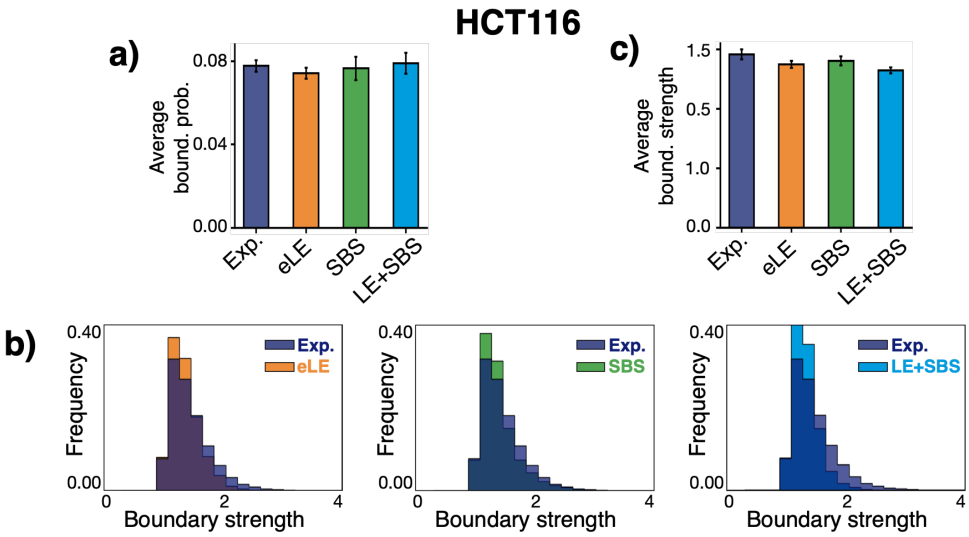

Supplementary Figure 9. The average boundary probability and strength are similar across the different models and close to the corresponding experimental values in HCT116.

a) TAD boundary probability averaged over all genomic positions in both experiment[1] and models in the HCT116 locus (error bars SEM). **b)** The distributions of the boundary strengths derived from the models are all similar to the experimental distribution, as well as **c)** their corresponding average values (error bars SEM). *n*=1000 independent single-molecule conformations for each model.

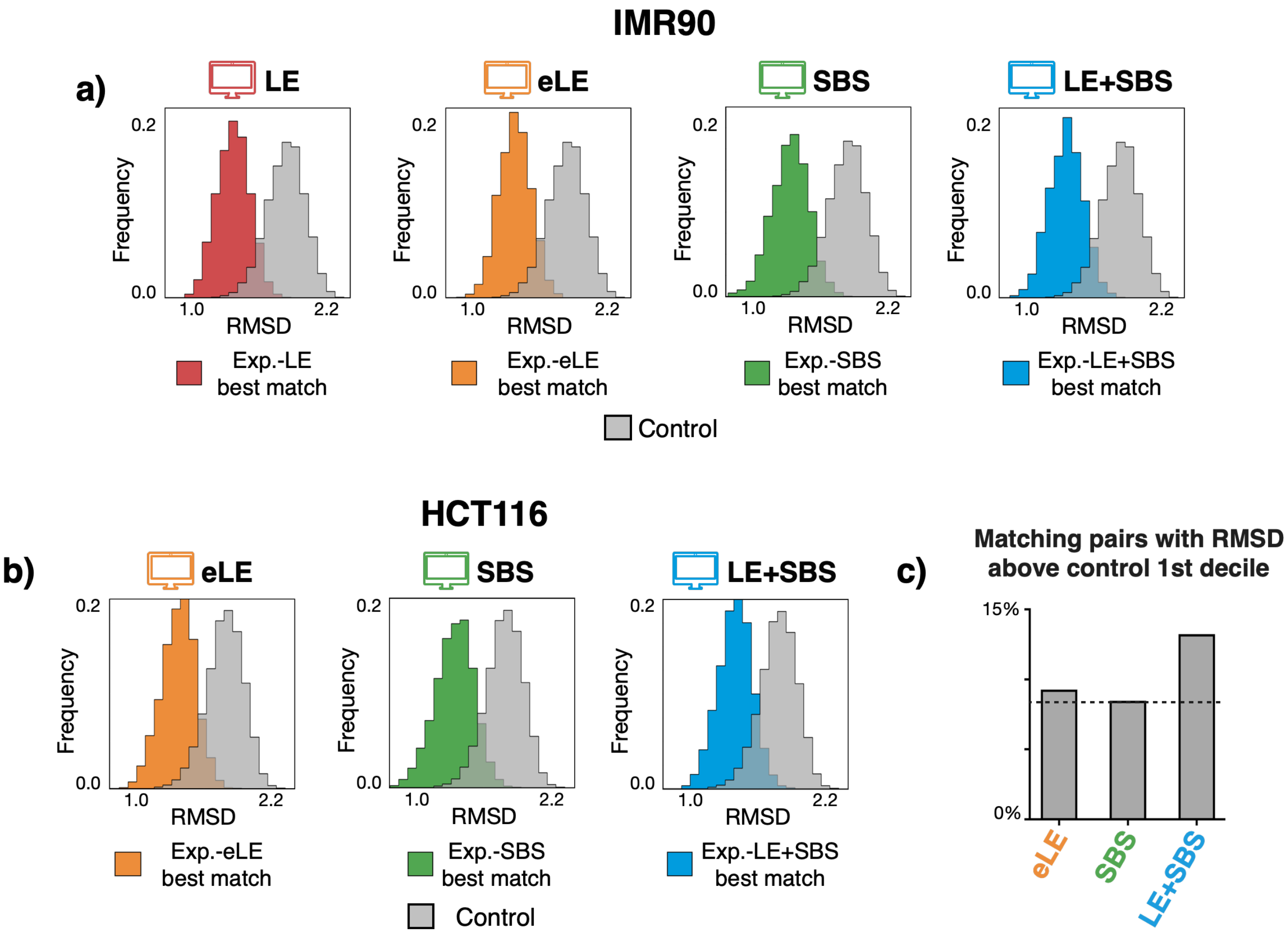

Supplementary Figure 10. The best-matching experiment-model pairs RMSD distributions are statistically different from a random control in the studied loci.

a) In the IMR90 cell locus, the RMSD distribution of the best-matching experiment-model pairs (for each of the considered polymer models) is statistically different from a control RMSD distribution made of random pairs of experimental structures (two-sided Mann–Whitney test p-value <0.001). **b)** Same as **a)** for the HCT116 cell locus. **c)** Less than 15% of the best matching pairs in the HCT116 locus have an RMSD above the 1st decile of the control distribution. The SBS, in particular, performs slightly better than the other models.

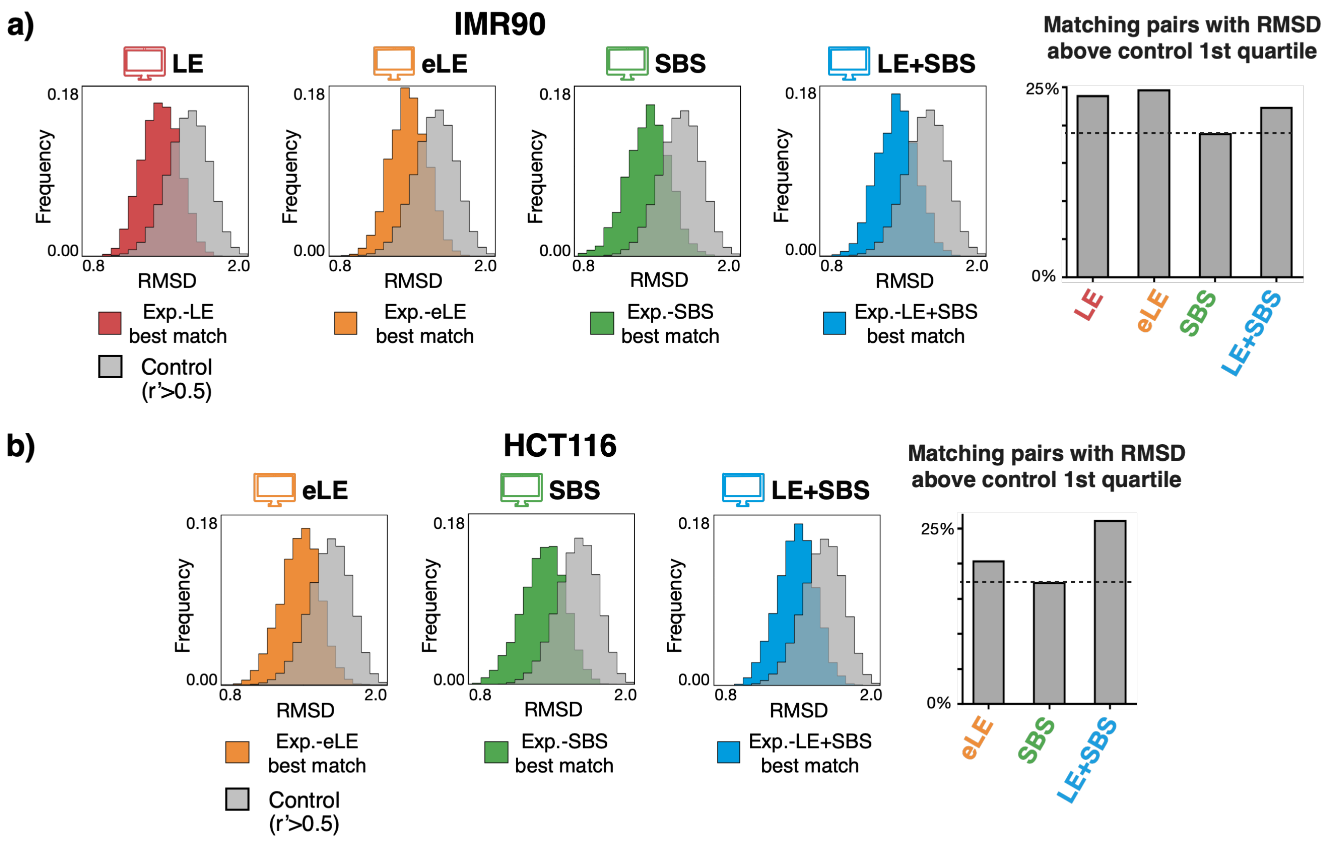

Supplementary Figure 11. An additional, more stringent control to test the statistical significance of the RMSD association provides analogous results.

To test the significance of the best-matching experiment-model pairs identified via the RMSD criterion, we performed an additional, more stringent control where the RMSD is computed only between pairs of microscopy conformations[1] having distance matrices with a corresponding genomic distance-corrected Pearson correlation value r'>0.5. We found that in both the **a)** IMR90 and **b)** HCT116 loci the RMSD distributions of the best-matches of each model are statistically different from the control (left, two-sided Mann–Whitney test p-value <0.001) with less than 25% of entries of the former falling above the 1st quartile of the latter (right). Hence, the model 3D conformations best matching the imaged structures have a statistically significant RMSD distribution and provide a non-trivial description of chromatin structure in single molecules.

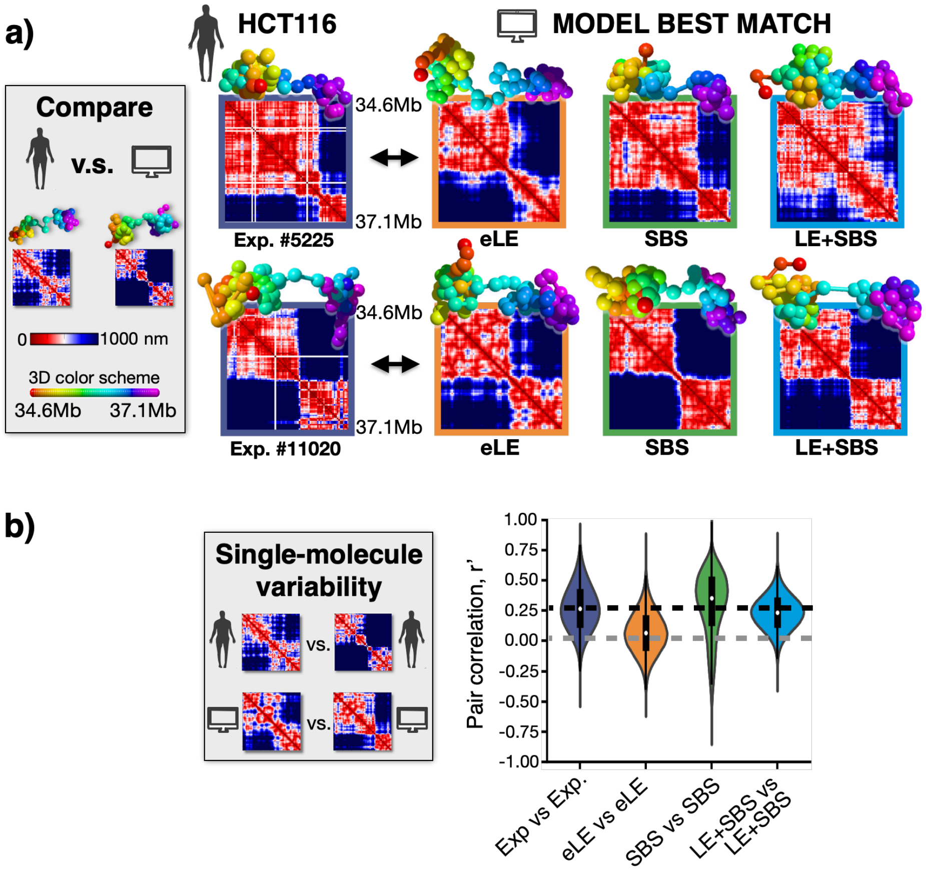

Supplementary Figure 12. Single-cell chromatin conformations of the HCT116 locus are well captured by the model 3D structures, especially by phase-separation based ones.

a) Microscopy single-cell chromatin structures of the HCT116 locus[1] (left) are associated to their best matching single-molecule conformation in each model via the minimum RMSD criterion. Two examples are shown here. **b**) The variability of single-cell imaged structures is measured by the distribution of r' correlations between pairs of distance matrices and is compared to the variability of in-silico structures. The experimental distribution is broad and has an average r'=0.27, while the corresponding average values from the models are: r'=0.07, r'=0.30, and r'=0.23, respectively, for the eLE, SBS, and LE+SBS. The boxplots represent the median, interquartile ranges, whiskers within 1.5 times the interquartile range. *n*=1000 independent single-molecule conformations for each model.

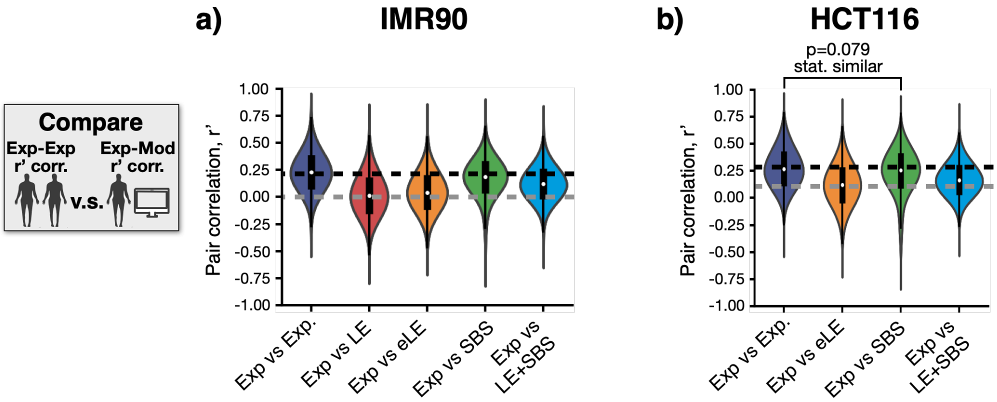

Supplementary Figure 13. Distributions of r' correlations between experiment and model single-molecule distance matrices.

a) The distribution of r' correlations between pairs of single-cell microscopy distance matrices[1] is compared, for each of the considered models, to the experiment-model r' distribution in the case of the IMR90 locus. **b)** Same as in panel **a)** for the HCT116 locus. In particular, the r' distribution in the case of the SBS model is statistically indistinguishable from the experimental one (two-sided Mann–Whitney test p-value = 0.079). The boxplots represent the median, interquartile ranges, whiskers within 1.5 times the interquartile range. *n*=1000 independent single-molecule conformations for each model.

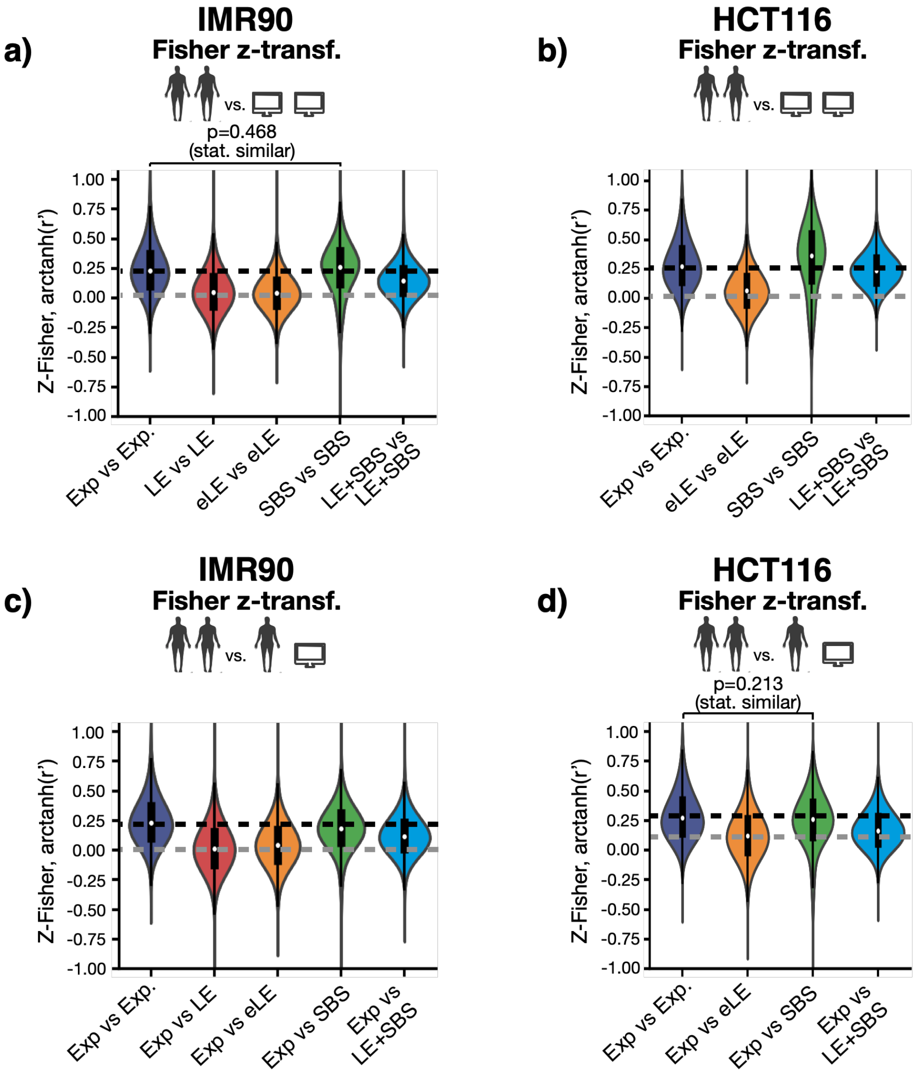

Supplementary Figure 14. Fisher z-transformation of r' correlations.

a) The distributions of the Fisher z-transformed r' correlations between exp-exp. and model-model single-molecule distance matrices are shown in the IMR90 cell locus. The Fisher z-transformed r' distribution of the SBS model is statistically indistinguishable from the experimental one (two-sided Mann–Whitney test p-value = 0.468). **b)** Same as **a)** for the HCT116 locus. **c)** The distributions of the Fisher z-transformed r' values between exp-exp. and exp-model single-molecule distance matrices are shown in the IMR90 case. **d)** Same as **c)** for the HCT116 case. In particular, the Fisher z-transformed r' distribution in the case of the SBS model is statistically indistinguishable from the experimental one (two-sided Mann–Whitney test p-value = 0.213). The r' and Fisher z-transformed r' distributions depict overall very similar scenarios. The boxplots represent the median, interquartile ranges, whiskers within 1.5 times the interquartile range. *n*=1000 independent single-molecule conformations for each model.

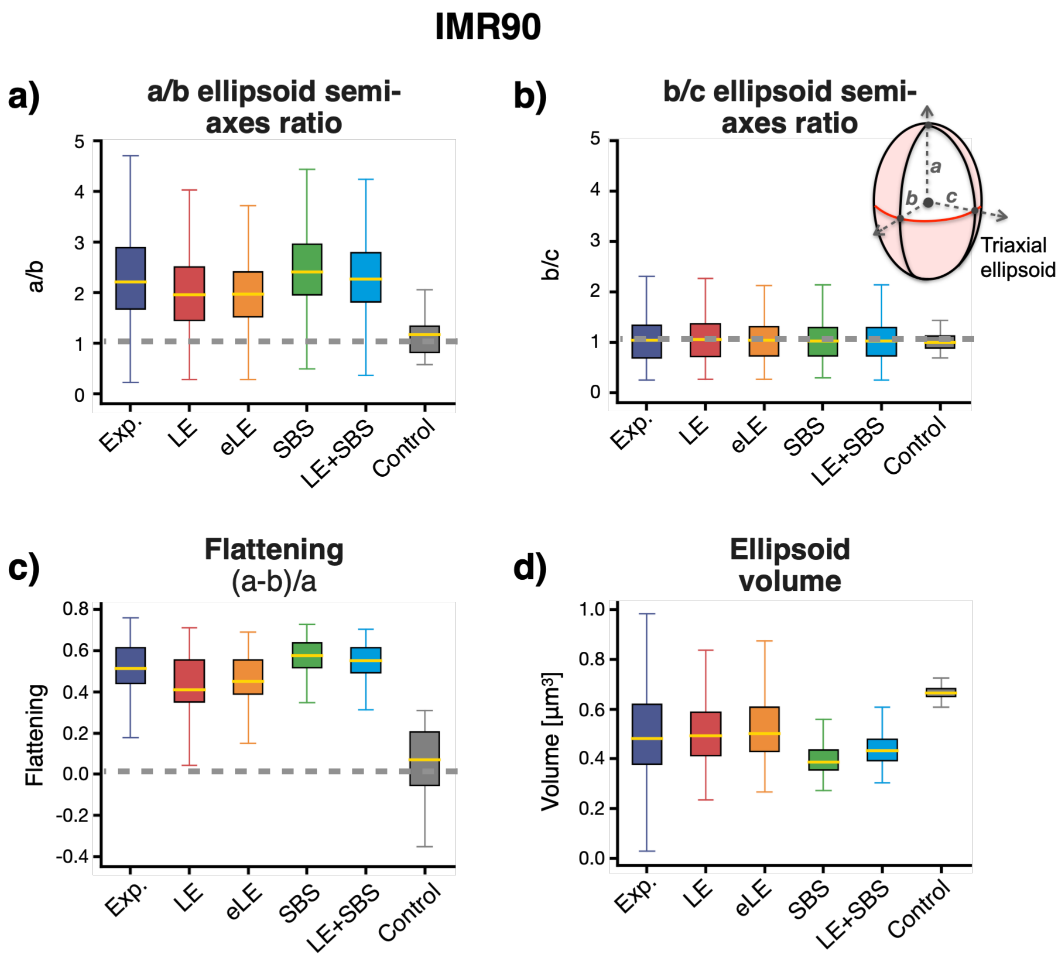

Supplementary Figure 15. Shape and volume factors of 3D single-molecule conformations in the IMR90 cell locus.

a) Comparison between experimental[1] and model derived distributions of the ellipsoid semi-axes ratio a/b. The control is a globule-like homopolymer with same number of beads and 3D size as the real images of the locus. **b)** Same as **a)** for the ellipsoid semi-axes ratio b/c. **c)** The distributions of the flattening parameter (i.e., $(a\text{-}b)/a$) show that, differently from the control, LE and phase-separation based models have a prolate shape consistent with microscopy[1] data (experimental average value 0.5). **d)** Volume distributions of the triaxial ellipsoid enclosing each imaged[1] and model single-molecule 3D structure (experimental average value 0.5μm^3). The boxplots represent the mean (yellow line), interquartile ranges, whiskers within 1.5 times the interquartile range. n=1000 independent single-molecule conformations for each model.

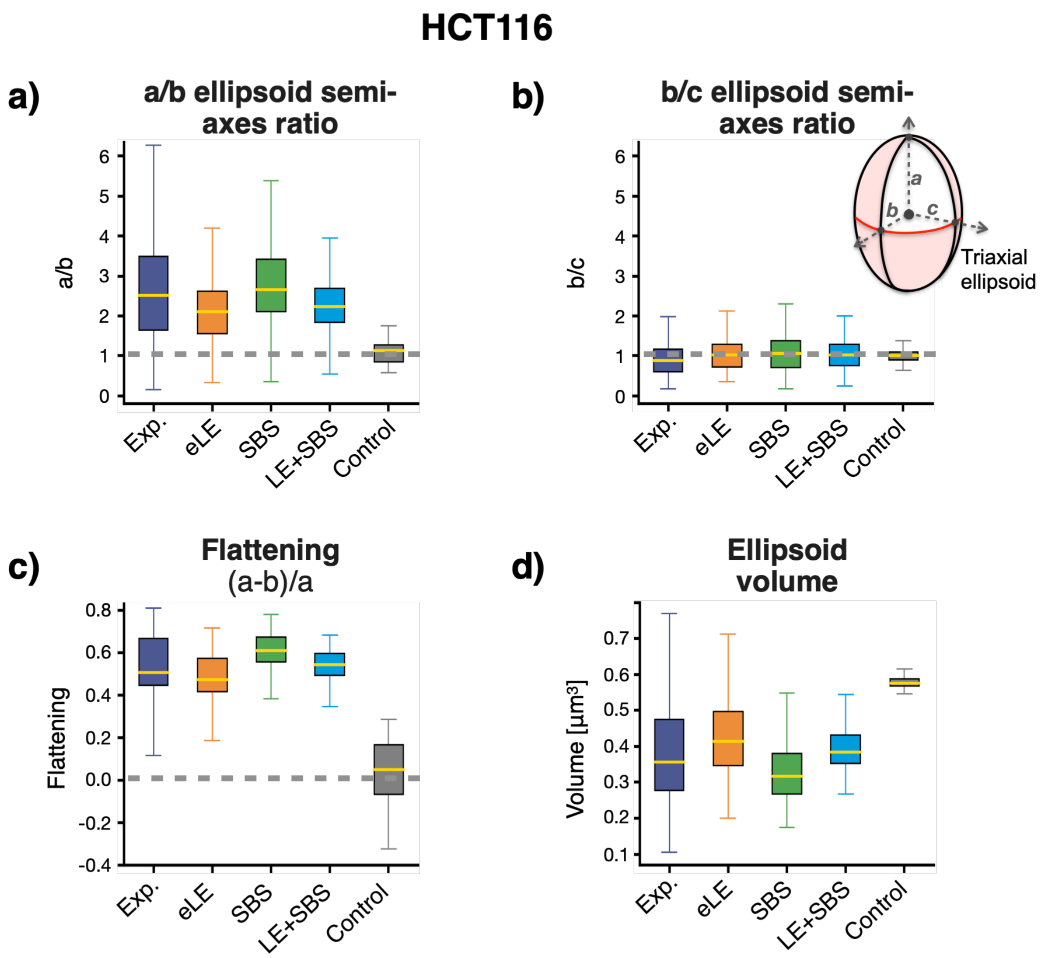

Supplementary Figure 16. Shape and volume factors of 3D single-molecule conformations in the HCT116 cell locus.

a) Comparison between experimental[1] and model derived distributions of the ellipsoid semi-axes ratio *a*/*b*. The control is a globule-like homopolymer with same number of beads and 3D size as the real images of the locus. **b)** Same as **a)** for the ellipsoid semi-axes ratio *b*/*c*. **c)** The distributions of the flattening parameter (i.e., (*a*-*b*)/*a*) show that, differently from the control, LE and phase-separation based models have a prolate shape consistent with experimental[1] data (experimental average value 0.5). **d)** Volume distribution of the triaxial ellipsoid enclosing each imaged[1] and model single-molecule 3D structure (experimental average value around 0.4μm^3). The boxplots represent the mean (yellow line), interquartile ranges, whiskers within 1.5 times the interquartile range. *n*=1000 independent single-molecule conformations for each model.

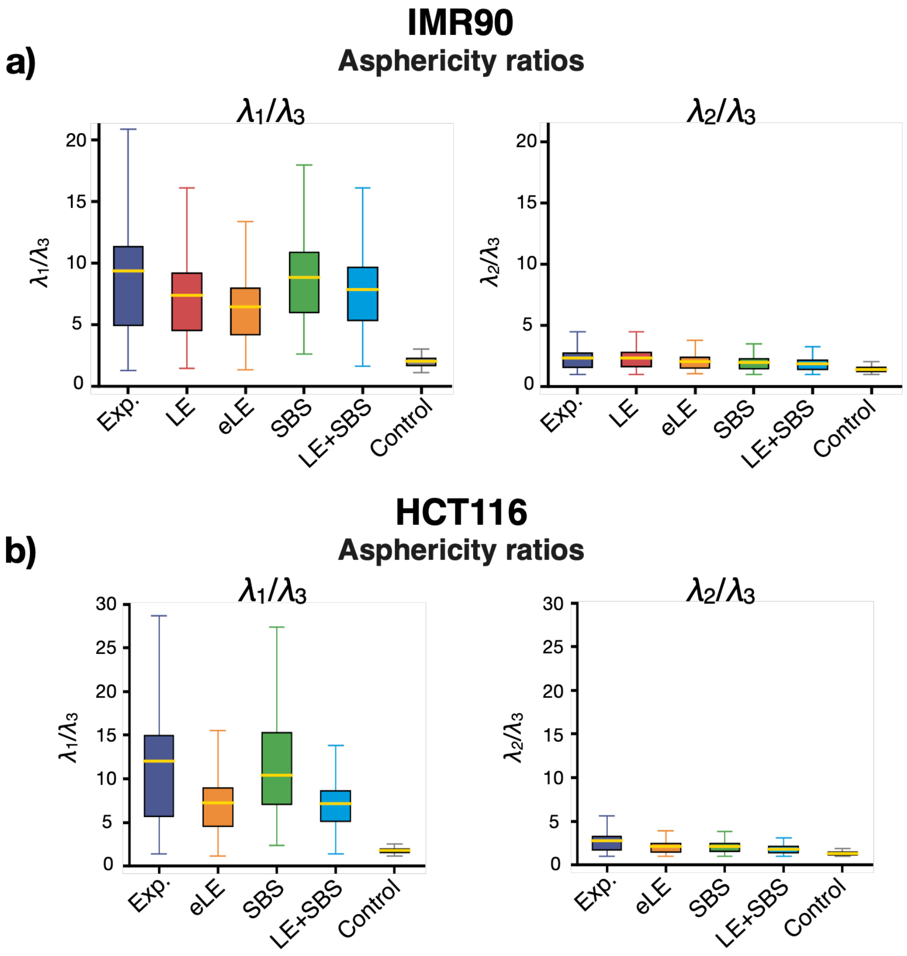

Supplementary Figure 17. Additional measures of shape factors of single-molecule conformations using the gyration tensor.

a) Asphericity ratios of single-molecule 3D structures measured by the eigenvalues of the corresponding gyration tensor in the IMR90 cell locus ($\lambda_3 \leq \lambda_2 \leq \lambda_1$). **b)** Same as **a)** for the HCT116 cell locus. Both imaged[1] and model single-molecule 3D conformations have a prolate shape. The boxplots represent the mean (yellow line), interquartile ranges, whiskers within 1.5 times the interquartile range. *n*=1000 independent single-molecule conformations for each model.

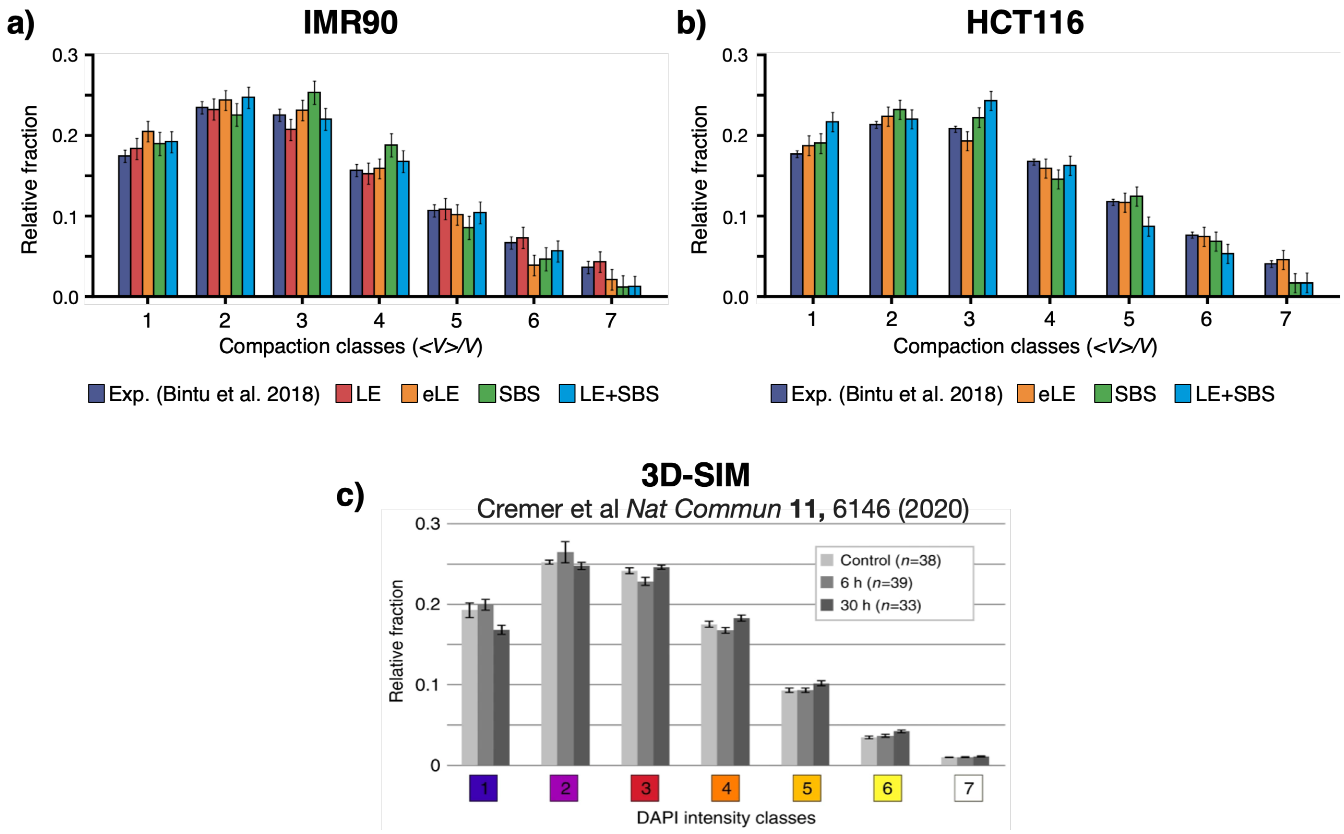

Supplementary Figure 18. Distribution of compaction classes across microscopy data and polymer models of chromatin.

a) Comparison of multiplexed microscopy data[1] and model derived distributions of chromatin compaction levels in the IMR90 cell locus (**Methods**). **b)** Same as **a)** for the HCT116 locus. *n*=1000 independent single-molecule conformations for each model. **c)** Relative 3D signal distributions of DAPI intensity classes in control and cohesin depleted nuclei (6 and 30 h, resp., incubation time in auxin); figure taken from[9]. The polymer models of our two studied genomic loci in human IMR90 and HCT116 cells, as well as multiplexed microscopy data from[1], match the distribution of chromatin compaction levels discovered by 3D-SIM across entire nuclei[9]. Data are presented as mean ± SEM.

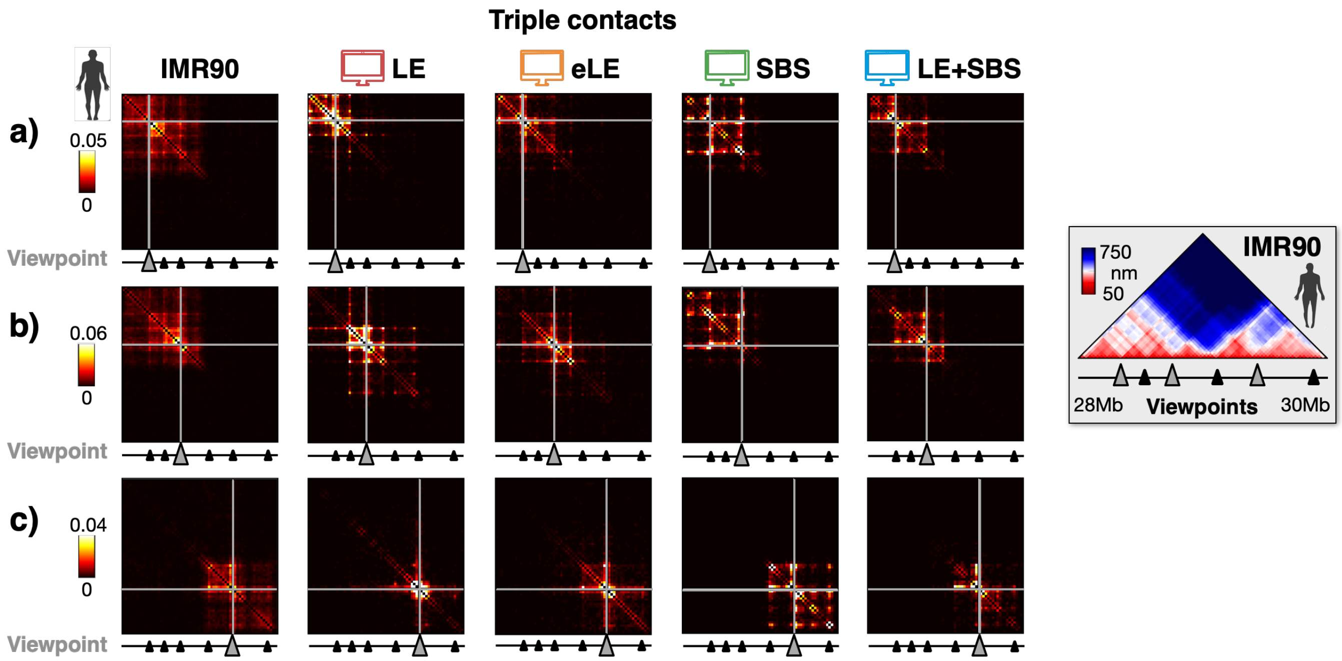

Supplementary Figure 19. Triple contact data from additional viewpoints in IMR90.

Triple contact probability maps are shown in microscopy data[1] (left) and in the models from three different, additional viewpoints in the IMR90 locus (panels **a)**, **b)**, **c)**). The experimental triplet contact patterns are well captured by the different models, especially by the eLE, SBS and LE+SBS. Source data are provided as a Source Data file.

SUPPLEMENTARY TABLES

a) IMR90

Pearson correlation, r
Models vs Exp.s

	Median distance	Hi-C contact	Boundary prob.	Separ. score
LE	r=0.90	r=0.87	r=0.31	r=0.51
eLE	r=0.93	r=0.95	r=0.83	r=0.74
SBS	r=0.96	r=0.94	r=0.63	r=0.79
LE+SBS	r=0.95	r=0.94	r=0.65	r=0.82

b) HCT116

Pearson correlation, r
Models vs Exp.s

	Median distance	Hi-C contact	Boundary prob.	Separ. score
eLE	r=0.94	r=0.97	r=0.50	r=0.62
SBS	r=0.95	r=0.88	r=0.79	r=0.85
LE+SBS	r=0.92	r=0.89	r=0.75	r=0.90

Supplementary Table 1.

The Pearson correlation coefficient, r, between model and experimental median distance[1] and Hi-C[6,8] contact maps provides results analogous to the genomic distance-corrected Pearson correlation coefficient, r', reported in the Main Text. The table also includes the r values of the comparisons between model and experimental boundary probability[1] and separation score[1], respectively, in the studied **a)** IMR90 and **b)** HCT116 human loci.

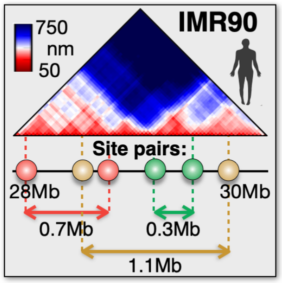

	a) Av. dist.+-st.d [nm]	b) Av. dist.+-st.d [nm]	c) Av. dist.+-st.d [nm]
	370+-190	860+-330	340+-200
LE	560+-220	750+-240	410+-160
eLE	510+-220	780+-260	360+-160
SBS	300+-130	880+-220	260+-90
LE+SBS	410+-150	840+-210	310+-100

Supplementary Table 2.

Average and standard deviation (in nm) of the physical distances of the IMR90 site pairs investigated in **Supplementary Fig. 6**.

REFERENCES

1. Bintu, B. *et al.* Super-resolution chromatin tracing reveals domains and cooperative interactions in single cells. *Science (80-.).* (2018) doi:10.1126/science.aau1783.

2. Dunham, I. *et al.* An integrated encyclopedia of DNA elements in the human genome. *Nature* **489**, 57–74 (2012).

3. Buckle, A., Brackley, C. A., Boyle, S., Marenduzzo, D. & Gilbert, N. Polymer Simulations of Heteromorphic Chromatin Predict the 3D Folding of Complex Genomic Loci. *Mol. Cell* **72**, (2018).

4. Conte, M. *et al.* Polymer physics indicates chromatin folding variability across single-cells results from state degeneracy in phase separation. *Nat. Commun.* (2020) doi:10.1038/s41467-020-17141-4.

5. Bianco, S. *et al.* Polymer physics predicts the effects of structural variants on chromatin architecture. *Nat. Genet.* (2018) doi:10.1038/s41588-018-0098-8.

6. Rao, S. S. P. *et al.* A 3D map of the human genome at kilobase resolution reveals principles of chromatin looping. *Cell* (2014) doi:10.1016/j.cell.2014.11.021.

7. De Gennes, P. G. Scaling concepts in polymer physics. Cornell university press. *Ithaca N.Y.,* (1979) doi:10.1163/_q3_SIM_00374.

8. Rao, S. S. P. *et al.* Cohesin Loss Eliminates All Loop Domains. *Cell* **171**, 305-320.e24 (2017).

9. Cremer, M. *et al.* Cohesin depleted cells rebuild functional nuclear compartments after endomitosis. *Nat. Commun.* **11**, (2020).
