# Supplementary material for: Loop-extrusion and polymer phase-separation can co-exist at the single-molecule level to shape chromatin folding
Source: Nat Commun. 2022 Jul 13;13:4070. doi: 10.1038/s41467-022-31856-6 (PMC9279381; doi:10.1038/s41467-022-31856-6)
Supplement: Supplementary file 2 — Reporting Summary [file 41467_2022_31856_MOESM2_ESM.pdf]

nature portfolio

Corresponding author(s): Mario Nicodemi

Last updated by author(s): Jul 1, 2022

Reporting Summary

Nature Portfolio wishes to improve the reproducibility of the work that we publish. This form provides structure for consistency and transparency in reporting. For further information on Nature Portfolio policies, see our Editorial Policies and the Editorial Policy Checklist.

Statistics

For all statistical analyses, confirm that the following items are present in the figure legend, table legend, main text, or Methods section.

n/a	Confirmed	
☐	☒	The exact sample size (*n*) for each experimental group/condition, given as a discrete number and unit of measurement
☐	☒	A statement on whether measurements were taken from distinct samples or whether the same sample was measured repeatedly
☐	☒	The statistical test(s) used AND whether they are one- or two-sided<br>*Only common tests should be described solely by name; describe more complex techniques in the Methods section.*
☒	☐	A description of all covariates tested
☒	☐	A description of any assumptions or corrections, such as tests of normality and adjustment for multiple comparisons
☐	☒	A full description of the statistical parameters including central tendency (e.g. means) or other basic estimates (e.g. regression coefficient) AND variation (e.g. standard deviation) or associated estimates of uncertainty (e.g. confidence intervals)
☐	☒	For null hypothesis testing, the test statistic (e.g. *F*, *t*, *r*) with confidence intervals, effect sizes, degrees of freedom and *P* value noted<br>*Give P values as exact values whenever suitable.*
☒	☐	For Bayesian analysis, information on the choice of priors and Markov chain Monte Carlo settings
☒	☐	For hierarchical and complex designs, identification of the appropriate level for tests and full reporting of outcomes
☐	☒	Estimates of effect sizes (e.g. Cohen's *d*, Pearson's *r*), indicating how they were calculated

Our web collection on statistics for biologists contains articles on many of the points above.

Software and code

Policy information about availability of computer code

Data collection	No software was used.
Data analysis	We used the publicly available LAMMPS (version 30july2016) and HOOMD-Blue software (version 2.9.x) for the Molecular Dynamics simulations of the polymer models. Analyses involving the computation of spatial distances were performed using built-in functions within the Python SciPy software (version 1.3.1). Positions and orientations of the model anchor sites were determined by a standard motif finding analysis using the FIMO tool within the MEME Suite software (version 5.4.1). The structural comparison of experimental and model conformations by RMSD was performed using the free available MDAnalysis Python library. We employed the POV-Ray software (version 3.7) to produce the 3D snapshots. All the codes required to perform the simulations of the polymer models are provided at https://github.com/ehsanirani/PhaseSeparation-LoopExtrusion-MD.

For manuscripts utilizing custom algorithms or software that are central to the research but not yet described in published literature, software must be made available to editors and reviewers. We strongly encourage code deposition in a community repository (e.g. GitHub). See the Nature Portfolio guidelines for submitting code & software for further information.

Data

Policy information about availability of data

All manuscripts must include a data availability statement. This statement should provide the following information, where applicable:

- Accession codes, unique identifiers, or web links for publicly available datasets
- A description of any restrictions on data availability
- For clinical datasets or third party data, please ensure that the statement adheres to our policy

The data supporting this study are available from the corresponding author upon request. Published Hi-C data [Rao et al. 2014; Rao et al. 2017] used for analyses

are available at the Gene Expression Omnibus (GEO) database with accession numbers GSE63525 and GSE104334. Published single-cell imaging data [Bintu et al. 2018] used in this study are available at https://github.com/BogdanBintu/ChromatinImaging. ChIP-seq data analyzed in this study were accessed via ENCODE (ENCODE accession for IMR90: ENCFF195CYT, ENCFF899APS, ENCFF474OJM, ENCFF752IXO, ENCFF178QVF, ENCFF741WIY, ENCFF625BTD, ENCFF448ZOJ, ENCFF732WRW, ENCFF453XKM; for HCT116: ENCFF391AAM, ENCFF899XEF, ENCFF711MPL, ENCFF931YSQ, ENCFF137TPC, ENCFF294LZM, ENCFF832IOO, ENCFF848IHI, ENCFF870WXZ, ENCFF463FGL). Source data are provided with this paper.

Field-specific reporting

Please select the one below that is the best fit for your research. If you are not sure, read the appropriate sections before making your selection.

☒ Life sciences ☐ Behavioural & social sciences ☐ Ecological, evolutionary & environmental sciences

For a reference copy of the document with all sections, see nature.com/documents/nr-reporting-summary-flat.pdf

Life sciences study design

All studies must disclose on these points even when the disclosure is negative.

Sample size	Our study is based on polymer physics computer simulations. No statistical methods were used to predetermine sample sizes. For each of the studied polymer models, we generated a statistical ensemble of n=1000 independent polymer conformations, which fully ensure robustness of statistical analyses.
Data exclusions	No data were excluded.
Replication	Our investigation is based on computer simulations and we do not generate new biological data. We verified the reproducibility of the findings of our manuscript running an additional battery of 200 independent numerical simulations for each studied locus.
Randomization	Randomization is not relevant to our study as in our work we performed polymer physics computer simulations and re-analyzed existing publicly available genomic datasets.
Blinding	Blinding is not relevant to our study as in our work we performed polymer physics computer simulations and re-analyzed existing publicly available genomic datasets.

Reporting for specific materials, systems and methods

We require information from authors about some types of materials, experimental systems and methods used in many studies. Here, indicate whether each material, system or method listed is relevant to your study. If you are not sure if a list item applies to your research, read the appropriate section before selecting a response.

Materials & experimental systems

n/a	Involved in the study	
☒	☐	Antibodies
☒	☐	Eukaryotic cell lines
☒	☐	Palaeontology and archaeology
☒	☐	Animals and other organisms
☒	☐	Human research participants
☒	☐	Clinical data
☒	☐	Dual use research of concern

Methods

n/a	Involved in the study	
☒	☐	ChIP-seq
☒	☐	Flow cytometry
☒	☐	MRI-based neuroimaging
